# The Global Health Impact Index: Promoting Global Health

**DOI:** 10.1371/journal.pone.0141374

**Published:** 2015-12-11

**Authors:** Nicole Hassoun

**Affiliations:** Department of Philosophy, Binghamton University, State University of New York, Binghamton, NY, United States of America; University College London, UNITED KINGDOM

## Abstract

Millions of people cannot access essential medicines they need for deadly diseases like malaria, tuberculosis (TB) and HIV/AIDS. There is good information on the need for drugs for these diseases but until now, no global estimate of the impact drugs are having on this burden. This paper presents a model measuring companies’ key malaria, TB and HIV/AIDS drugs’ consequences for global health (global-health-impact.org). It aggregates drugs’ impacts in several ways–by disease, country and originator-company. The methodology can be extended across diseases as well as drugs to provide a more extensive picture of the impact companies’ drugs are having on the global burden of disease. The study suggests that key malaria, TB and HIV/AIDS drugs are, together, ameliorating about 37% of the global burden of these diseases and Sanofi, Novartis, and Pfizer’s drugs are having the largest effect on this burden. Moreover, drug impacts vary widely across countries. This index provides important information for policy makers, pharmaceutical companies, countries, and other stake-holders that can help increase access to essential medicines.

## Introduction

Many people around the world cannot access essential medicines to treat diseases like malaria, tuberculosis (TB), and HIV/AIDS. Every year 8.8 million people are diagnosed with tuberculosis, every day more than 7,300 people are infected with AIDs, every 60 seconds malaria kills a child [1–3]. One reason for this is that many people cannot access the existing drugs and technologies they need. Even basic medicines, like antibiotics, may be too expensive for people in low income countries making less than the equivalent of what $1,025 a year will buy in the US [4]. Another reason many people around the world lack access to essential medicines is that little of the research and development on new drugs and technologies focuses on the diseases that have the largest impact on global health [5].

There are several ways of trying to address these problems. Many global health organizations including the Global Fund offer grants for developing countries to purchase essential medicines or provide these directly to the poor. Others, like PATH, GAVI, IPM Global, and the TB Alliance, partner with private industry to do research on, and development of, essential medicines [6]. Yet others, like the Gates Foundation and the US Food and Drug Administration, support these efforts by offering financial incentives for new innovation in the form of grants, prize funds, priority review vouchers, and advanced market commitments [7]. Better data on everything from health systems functioning, and administration to the impact of essential medicines is necessary to advance these efforts [8].

Some good data is already available and having a large impact. The Institute for Health Metrics Evaluation’s Global Burden of Disease project provides estimates of the need for many essential medicines, for instance [9]. This project estimates the Disability Adjusted Life Years (DALYS)–a measure of death and disability lost–due to many of the major global health problems in each country in the world.

Until now, however, we have had no consistent estimates of the impact of our efforts to combat malaria, TB, and HIV/AIDS. The Institute for Health Metrics Evaluation does not provide any estimate of how much of this burden is alleviated across these diseases. The models that do exist focus primarily on individual diseases [10–12]. This paper presents a new Global Health Impact index that evaluates the impact of key drugs for malaria, TB, and HIV/AIDS around the world.

## Materials and Methods

What is the best way to measure the global health impact of key medicines for some of the world’s worst diseases? Is it possible to develop a consistent and simple method of measuring the death and disability averted by essential medicines?

**Fig 1:**
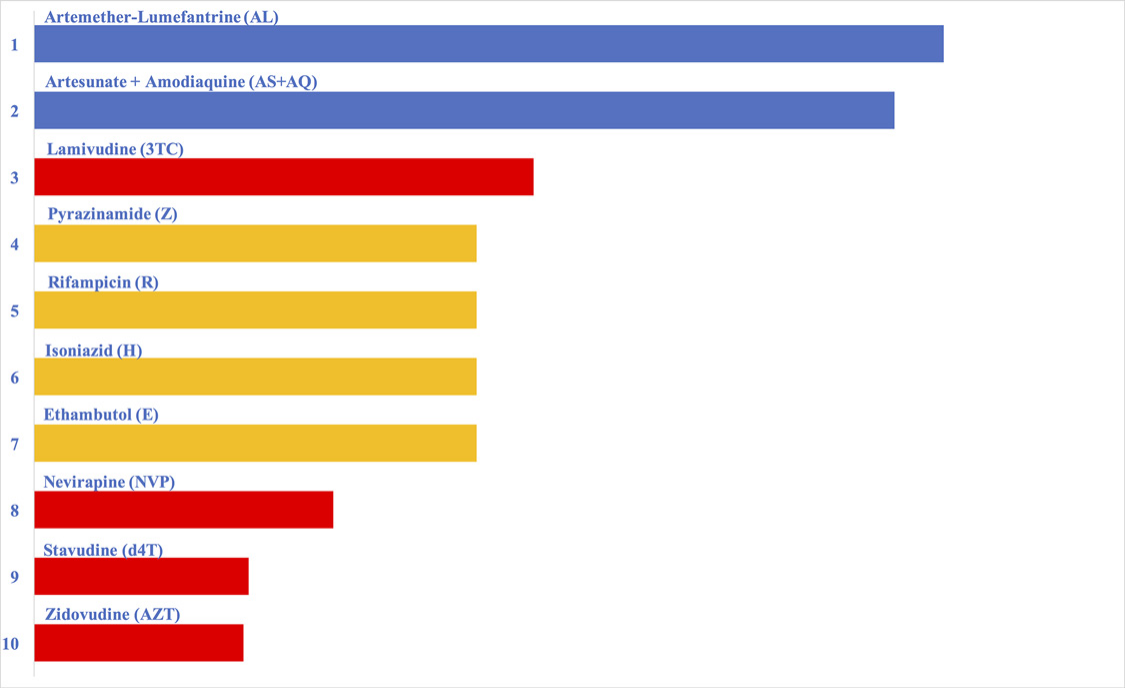
Impact scores of individual drugs by rank. A graph of the comparative impact of the top 10 drugs ranked from 1 (highest impact) to 10 (lowest impact). Blue bars represent malaria drugs, yellow bars represent TB drugs, and red bars represent HIV drugs.

The Global Health Impact Index uses three pieces of information in arriving at an estimate of key drugs’ impact on malaria, TB, and HIV/AIDS around the world: data on access to the drugs, their efficacy and the need for those drugs (measured in DALYS) [9]. The base year for the model is 2010 and the impact of drug *i* for disease state *j* in the base year in each country *c*, is calculated as

$${I_{icj}=d}_{icj}t_{icj}e_{icj}.$$

where *d*_*icj*_ represents the DALYS lost to disease state *j* in country *c* that we estimate can be averted with *i*; *t*_*icj*_ represents the proportion of people who need treatment for disease state *j* who we estimate are receiving *i* in country *c*; *e*_*icj*_ represents estimated treatment effectiveness of *i* in *c* for disease state *j*. Each drug’s score is the sum of its scores in each country for all disease states (i.e. the sum of scores for drug susceptible TB in HIV+/- patients, multi-drug resistant, and extremely drug resistant TB). Fig 1 shows drug scores by rank and details about this are provided in S1 Appendix [13–28]. Because each drug’s impact varies with the need for, access to, and efficacy of the medicine this model provides much more than a scientific study of drugs’ efficacy or meta-analysis. It looks at the proportion of the population that needs the medicine that is receiving it as well as drug efficacy. It calculates the proportion of the global burden of disease alleviated.

**Fig 2:**
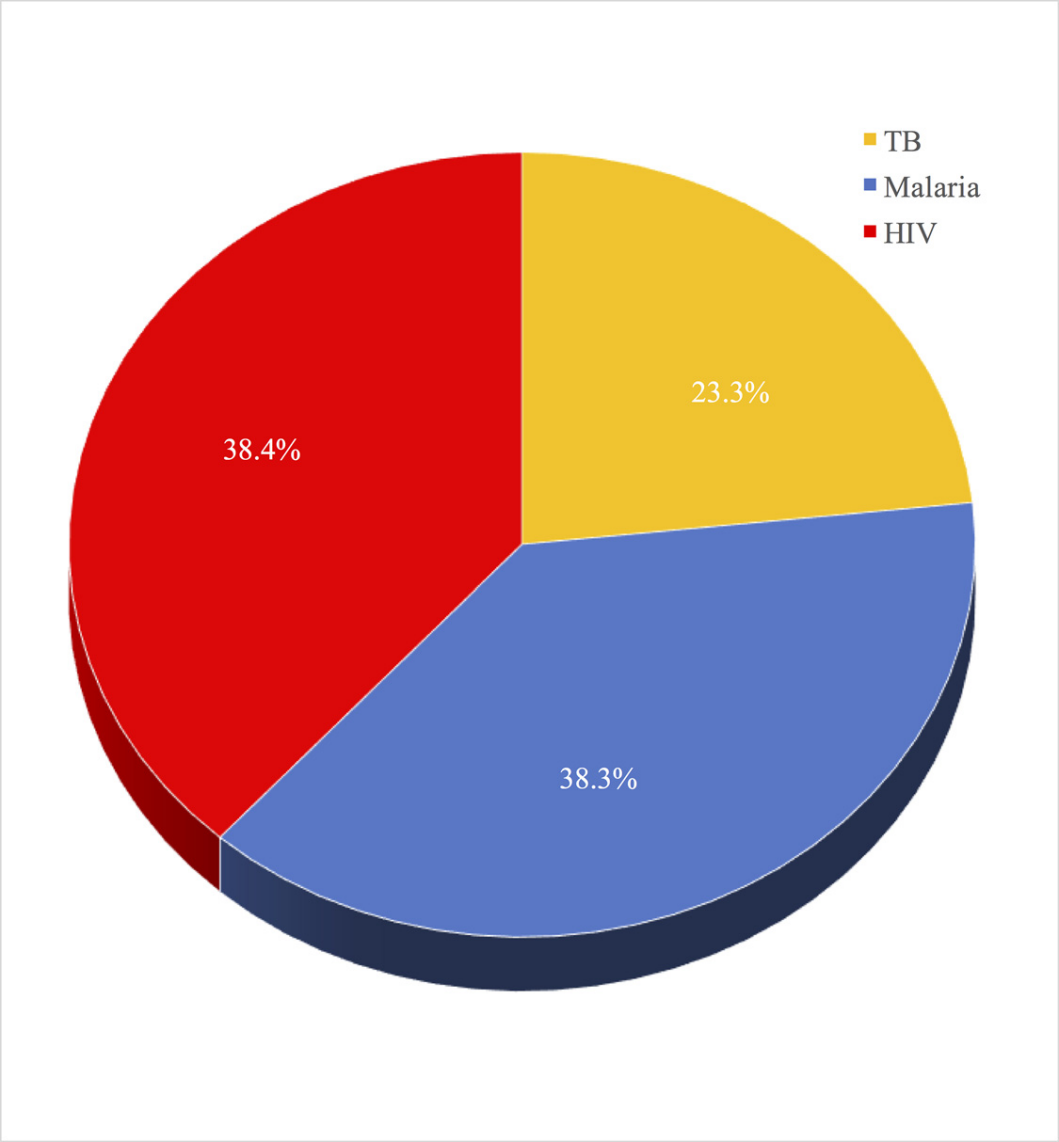
Total impact scores for drugs aggregated by disease. What proportion of aggregate drug impacts is attributable to Malaria, TB, and HIV.

Individual drug scores can be aggregated in many ways–within countries, across countries, by disease and even by pharmaceutical company holding the patent on the medicines. It is possible to see, for instance, which drugs are alleviating the largest amount of death and disability in each country in the world and, where great needs remain unmet, and to what extent this is due to lack of access to effective drugs. As Fig 2 demonstrates, it is also possible to aggregate drug scores by disease. All of the data in the index is from international databases and reports (see S1 Appendix for further explanation) [14–28]. Where country-level information is not available, our approach is to use averages to replace the missing national data.

It will help to consider a real example of how one originator company’s drug’s score was calculated. Chongqing Tonghe Pharmaceutical Co. Ltd. holds the patent on only one anti-malarial: Dihydroartemisinin-piperaquine (DHA-PPQ). DHA-PPQ is a first-line drug in Viet Nam, so consider how its impact in Viet Nam was calculated. 63,901.40 DALYs were lost to malaria in Viet Nam in 2010. About 75% of the malaria in Viet Nam was Plasmodium falciparum malaria [9]. so we estimate that 75% of the 63,901.40—or 47,926.05—DALYs were lost to p. falciparum malaria. DHA-PPQ is used as the first-line treatment of Plasmodium falciparum malaria in Viet Nam. The WHO suggests that DHA-PPQ is 100% effective in Viet Nam. However, DHA-PPQ only has treatment coverage in Viet Nam of 2.6% [9, 29–32]. The impact of DHA-PPQ in Viet Nam, then, is DALYs * Coverage * Effectiveness. The estimated impact of DHA-PPQ for Viet Nam is (47,926.05 * 2.6% * 100%) = 1,246.08 DALYs saved. The above process was repeated for every country where DHA-PPQ was a first-line drug, so that an impact score for every country was obtained. To get the total impact score for Chongqing Tonghe Pharmaceutical Co. Ltd., we summed the scores for all of these countries. The total impact score for Chongqing Tonghe Pharmaceutical Co. Ltd. is 6,730.11 DALYs saved. The models used to calculate TB and HIV are much more complex, so examples illustrating the necessary calculations are relegated to the S1 Appendix Sections II and III. What follows just presents some key assumptions and data sources for our TB and HIV models in turn.

Consider our current TB model. It examines drug impacts on Drug-Susceptible (or "Normal") TB, MDR-TB, and XDR-TB. Totally drug-resistant (TDR)-TB is excluded from the current model as, thus far, only a small number of TDR-TB cases have been reported [14]. For Drug-Susceptible TB, we also consider the difference in drugs’ impacts on HIV+ versus HIV- TB cases. UNAIDS’ TB database provides the data necessary for breaking down incident cases into Drug-Susceptible TB and MDR-TB, and we use the WHO’s Global Tuberculosis Report’s estimate that 9% of MDR-TB cases are XDR-TB [18]. The WHO reports the number incident cases of TB, treatment percentages, the number of registered cases that were tested for HIV status, and what proportion were HIV positive [16, 19, 20]. In countries where data is not available regarding the proportion of TB incident cases with known HIV status, an estimate was derived using the global average percent of TB cases with a known HIV status [21]. The Institute for Health Metrics Evaluation provides data on the DALYS lost to TB [9]. To compute the DALYs lost to MDR-TB and XDR-TB, we use calculated MDR-TB and XDR-TB proportions and multiply by the total DALYs due to TB of any type. We use global estimates of drug efficacy for the various treatment groups (e.g. MDR-TB) [17]. We have yet to get good treatment coverage data at the country level for HIV+ vs HIV- drug susceptible TB cases. Thus, for now, we use the WHO’s estimate of the prevalence of directly observed treatment short-course (DOTS) coverage of 65.9% for all cases [21]. Estimated efficacy for TB/HIV+ treatment is 73% and that for TB/HIV- treatment is 87% [17].

We then take the calculated DALYs lost to each specific type of TB (e.g. Normal TB in HIV+ patients) * treatment coverage for that type with each regimen * efficacy of treatment for that type in each country to get regimen impact scores. Each drug in the regimen is given credit in proportion to its use. An individual drug’s score, then, is the sum of each of the proportional score of any regimen in which it is a part. Drug scores are aggregated across countries and then across disease type and by originator company.

A difficulty that arises with MDR-TB treatment is that different patients are resistant to different drugs. Protocols exist for which treatment to use depending on to which drugs the patient is resistant. We use these protocols and data on resistance to estimate global use of each regimen [33, 34]. Again, each drug in each regimen is given equal weight in our model.

Next, consider our HIV model. Again, the Institute for Health Metrics Evaluation provides data on the DALYS lost to HIV [9]. Treatment percentage information is available from the WHO’s HIV database [24]. If country-specific treatment numbers are not provided, then numbers are calculated based on what would be necessary to reach regional averages [25, 26]. The WHO also provides data on what percentage of adults and children are taking first and second line regimens in mid- and low-income countries affected by HIV that responded to the WHO AIDS Medicines and Diagnostics Service (AMDS) survey. These countries were classified by the WHO as either "Group A" or "Group B" countries. Group A countries include 47 low- and middle-income countries excluding region of the Americas and Group B countries include 20 low- and middle-income countries in the Americas [27]. We assume that the DALYs each regimen can recover are proportionate to their use in each population. We conducted a systematic review of regimens’ efficacy by subgroup (e.g. children in Group B countries) using data on the regimen from other regions and then data on average regimen efficacy within subgroups as fallback data when efficacy estimates were not available [35–69].

**Fig 3:**
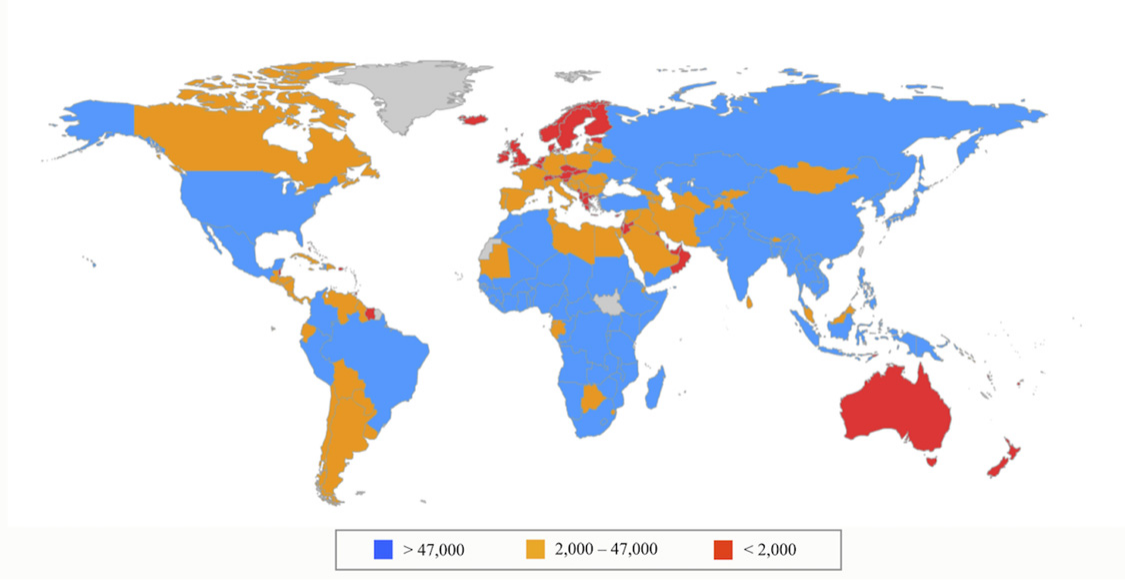
Estimated Disability Adjusted Life Years averted in each country. A graph showing how many DALYs are averted globally, divided by country.

We take the calculated DALYs lost recoverable with a particular treatment regimen in a country for each subgroup of patients (e.g. Group A adults) * treatment coverage for that subgroup in that country with each regimen * estimated efficacy of treatment for that subgroup to get regimen impact scores. Each drug in the regimen is given credit in proportion to its use. An individual drug’s score, then, is the sum of its proportional of each regimen’s score in which it is a part. Drug scores are aggregated across countries (as in Fig 3) and then across disease type and by originator company.

We have conducted extensive one-way and multi-parameter sensitivity analysis on the model as well as Monte Carlo analysis. Overall the model is stable across a wide range of parameter settings and changes to modelling assumptions. Again, much more detailed explanations with example calculations for TB and HIV and details of some the sensitivity analyses are available in the S1 Appendix. Additional results are available from the corresponding author upon request.

There are important limitations to the quality of the data we use. Our analysis does not, for instance, perfectly capture the volume of each product used in each country for all diseases. Where data is lacking, we provide an estimate based on the extent to which products are listed as first-line drugs for treating a disease. If 100 patients are treated in a country, and there are two first-line drugs, we give each drug credit for its potential impact on 50 patients. Our approach has the merit of allocating credit for being recognized as a first-line therapy for a given disease. Similarly, we have not attempted to determine what the incremental benefit of a drug is, relative to the next best therapy. Our analysis, in effect, indicates the impact of treatment including drugs in place of no treatment. Details of some sensitivity analyses on the model are included in S1 Appendix Section IV. Even with the inaccuracies that inevitably arise in this exercise, we believe that it is important to estimate the impact pharmaceuticals are having on global health for patients infected with malaria, tuberculosis and HIV/AIDS.

In the future, we plan to expand the model over time and across diseases. There is good data, for instance, on a variety of neglected diseases that have a large global health impact including lymphatic filariasis, helminths, and schistosomiasis. It may also be possible to expand the index to include some chronic diseases and different emerging technologies including vaccines.

## Results

The model provides an estimate of the impact drugs for malaria, TB, and HIV are having around the world. Key drugs for malaria–artemether-lumefantrine and artesunate plus amodiaquine–have the largest impact because they are widely recommended and highly effective. Together they alleviate almost 12% of the global burden of the three diseases in the model. Lamivudine for HIV and all of the drugs in the first line regimen for drug susceptible TB also have a large impact. Still, even with many highly effective drugs available, 63% of the burden of these diseases remains unalleviated.

**Fig 4:**
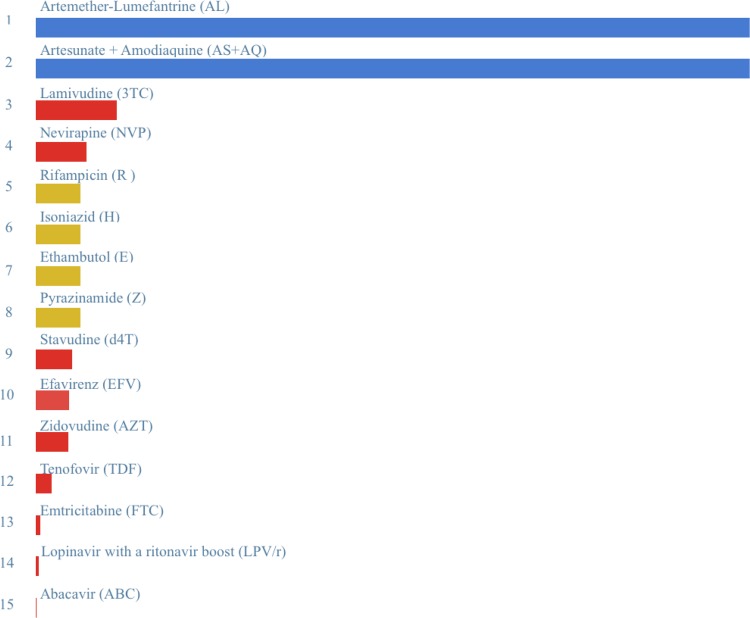
Impact scores of individual drugs by rank for Nigeria. A graph of the comparative impact of 15 drugs ranked from 1 (highest impact) to 15 (lowest impact) for the treatment of Malaria in Nigeria. Blue bars represent malaria drugs, yellow bars represent TB drugs, and red bars represent HIV drugs.

It is also possible to see the drugs’ impacts in each country individually, by disease, and in aggregate. Nigeria is the highest ranked country on the Global Health Index. There is a lot of malaria in Nigeria receiving highly effective treatment (See Fig 4). There is also some successfully treated malaria and TB. In India drugs for TB are having a much larger impact, though we are also having some significant impact on HIV/AIDS. In South Africa, the third highest ranked country, we are having more success in alleviating the burden of HIV/AIDS but there is a much less severe malaria burden to alleviate.

Moreover, the model provides an overall picture of drugs’ impacts on these three diseases and it is possible to view the determinants of drug performance in the model many ways. The model suggests, for instance, that we are having an approximately equal impact on all three diseases, though the need for drugs for TB is much less than for malaria or HIV/AIDS, treatment coverage is higher. There is, however, great variation across countries and regions in need, treatment coverage, and efficacy. Treatment coverage for HIV/AIDS is significantly higher in Latin America and the Caribbean than many other regions, for instance.

Finally, the model shows the drugs impacts aggregated by originator-company. The model suggests that drugs on which Sanofi holds the original patent or license are having the greatest impact of any company’s drugs. Most of the impact comes from its drugs for malaria (artesunate plus amodiaquine and artesunate plus sulfadoxine-pyrimethamine). These drugs are responsible for 50.07% of the total DALYs alleviated that would have been lost to malaria in the world in 2010. Sanofi also has the original patent on some key medicines for TB (Rifampicin and Ethionamide). Rifampicin is important because it is one of the standard 6-month first-line regimens against TB. Ethionamide is especially important in treating multiple drug-resistant TB (MDR-TB) because it is included in all three anti-MDR-TB treatment regimens. Sanofi’s TB drugs helped to alleviate 24.97% of the DALYs that would have been lost to TB in the world of 2010 [27].

## Discussion

The Global Health Impact Index can assist ministries of health and country-level policy makers in developing health policy. It may be useful for country-level health ministries in deciding what diseases to prioritize and what drugs to use. The claim here is not that the best drugs in the model are the ones countries should use. Nor is it obvious that we should put more resources into fighting the diseases against which we are most successful. Countries should still, presumably, follow WHO treatment guidelines and may do best to focus on meeting the greatest needs. Nevertheless, when the suggested drugs, or efforts to address a disease, are not significantly impacting death and disability, policy makers should consider why–there are many possible explanations including price differentials, resistance patterns, and so forth. The model shows, for instance, that some highly effective HIV/AIDS drugs are not having a large impact because they are not widely used. Countries might draw on this fact to explain the potential impact of scaling up such treatments in applying for grants from organizations like the Global Fund. Alternately, they could compare the impact of key drugs within their country to countries with relevantly similar disease burdens etc. Nigeria might, for instance, point out that despite great remaining need for anti-malaria drugs, drugs are having a large impact in combatting the disease within their borders. On the other hand, policy makers can inquire into why drugs in India do not seem to be having a very large impact on malaria despite the large need (especially given that many countries with similarly high disease burdens–like Uganda—are much more successful in addressing the need).

The model can also be used to evaluate international organizations’ contributions to global health impact and to help them focus their efforts more effectively. Generally, these organizations claim a proportion of the total impact of interventions in partner-countries. This model has some advantages over, e.g., the Global Fund’s method of estimating the impact of its investments [70]. It is simple, transparent, and consistent across several diseases and can be extended to evaluate a broad range of interventions. It also includes a measure of the disability as well as death averted by key interventions.

It is important to recall, however, that a drug’s impact varies with the need for, access to, and efficacy of the medicine. Some drugs may be available, acceptable, affordable and accessible, but ineffective. Others may cost too much, or the company that produces them may not be able to make them in sufficient quantities, to have a great impact even if they are highly effective. In yet other cases, prices may rise as demand increases–limiting accessibility. This may happen if, for instance, policy makers include a drug in treatment guidelines without international organizations or countries investing sufficiently in scale-up. In some cases, even climate change can explain changes in impact scores as rainfall, for instance, impacts the transmission of malaria. Not all factors that contribute to impact result from countries’, companies’, or international organizations’ attempts to extend access on essential medicines (some have nothing to do with drugs at all). However, the index opens the door to figuring out what factors are important determinants of impact in different contexts. Policy makers may look for correlations between policies and impacts to determine what factors promote and undermine global health impact in general and design better policy. They can also try to address the particular problems they discover.

**Fig 5:**
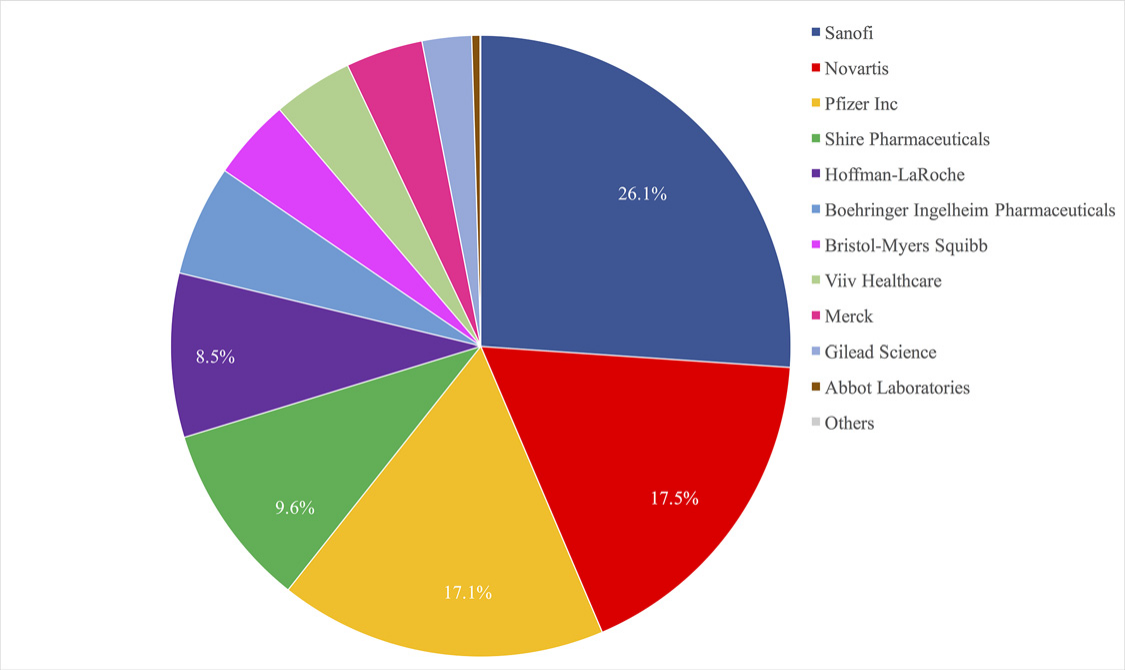
Aggregated drug impact scores for each company. The proportion of total impact attributable to companies based upon the drugs they have originated.

Further research using the index will allow us to have a clearer picture of the causes and consequences of this impact. Pharmaceutical companies, consumers, and investors may utilize this index to track performance, set targets, and create incentives for greater impact. In other work, I have argued for using this kind of index as a basis for giving companies a Global Health Impact label to use on all of their over-the-counter products. If even a small percentage of consumers prefer products with Global Health Impact labels, the incentive for companies to improve performance may be significant [35]. Again, companies are not the only ones responsible for the drugs’ impacts. The Drugs for Neglected Diseases Initiative (DNDi) played a key role, for instance, in the development of artesunate+sulfadoxine-pyrimethamine. Still, as discussed above, aggregating drug scores by the companies holding the patents/licenses (as in Fig 5) provides a mechanism for incentivizing new innovation.

Moreover, the company or country indexes are in no way an overall evaluation of companies’ or countries’ performance or even their access policies. Many things affect drug performance including other countries’, companies’, and international institutions’ policies. Right now we just aggregate drug scores by originator-company (it was difficult even to determine which companies held the original patents on which medicines). In the future, we plan to look at manufacturers and distributors as well. This is incredibly important as studies indicate that Indian generic manufacturers supply more than 80% of ARVs in low- and middle-income countries and their efforts are essential for lowering prices and increasing drug accessibility [71]. However, we have not yet perfected our methodology for doing so and do not present the data here as the Global Price Reporting Mechanism Database does not (yet) contain information about distributions of anti-malarial medicines (some preliminary data is, however, available upon request from the corresponding author). Nevertheless, we think it is illuminating to see which originator companies’ drugs are having an impact, and where they are having an impact, as this provides a mechanism for incentivizing positive change.

## Conclusions

There are many difficult issues to resolve in evaluating key innovations’ impact on global health. It is possible to refine many of the assumptions the model relies upon further and to update the model as new data on need, access, and efficacy becomes available. Nevertheless, the model presented here provides some essential information about drugs’ global health impacts and highlights the need to improve global disease surveillance mechanisms. A good rating system has the potential to foster great improvements to global health. The rating system should be of interest to policy makers, researchers, companies, investors, and consumers. It will, for instance, open the door to many new ways of incentivizing international organizations, countries, and companies to have a greater impact on global health. Although this will not solve all of the health problems people face, it may help many people secure essential medicines that can save millions of lives every year.

## Supporting Information

S1 Appendix Drug Impacts, Sensitivity Analyses, and Company Accreditation. Contents:
Section I. Drug Abbreviations. (p. 2) Table A, Drug Abbreviations.
Section II. TB Example: How the Impacts of TB Drugs are Calculated. (p. 3)
Fig A, Breakdown of TB patient groups. Table B, Impact on TB patient groups for Botswana in 2010. Table C, Disaggregated Drug-Susceptible TB Treatment Regimen. Table D, Possible MDR-TB Treatment Regimens. Table E, MDR-TB Resistance Patterns. Table F, Breakdown of TB Drug Resistance between Different Regimens. Table G, Portion of MDR-TB treatments. Table H, Proportion of Credit Given to Each Drug in Different Treatment Regimens. Table I, XDR-TB Treatment Regimen Drug Proportions. Table J, Disaggregated XDR-TB Treatment Regimens.
Section III. HIV Example: How the Impacts of HIV Drugs are Calculated. (p. 11)
Table K, Group A and Group B Countries Affected by HIV. Table L, Percentage of People Taking First and Second Line Regimens in Group A Countries. Table M, Antiretroviral Treatment Regimen Proportions and Efficacies for Group A and Group B Countries. Some of the material in this section is reprinted from “Globalization, Global Justice, and Global Health Impact” under a CC BY license, with permission from Public Affairs Quarterly, original copyright 2014
Section IV. Sensitivity Analyses. (p. 18)
Fig B, Initial and Secondary Stability for Sensitivity Analyses.
Section V. Drug Accreditation between Companies. (p. 25)
Table N, Drug Accreditation between Companies.
(DOCX)
