# Correction: The Global Health Impact Index: Promoting Global Health

**DOI:** 10.1371/journal.pone.0148946

**Published:** 2016-02-05

**Authors:** Nicole Hassoun

The captions for Figs 2 and 3 are incorrect. The correct, complete captions are:

**Fig 2:**
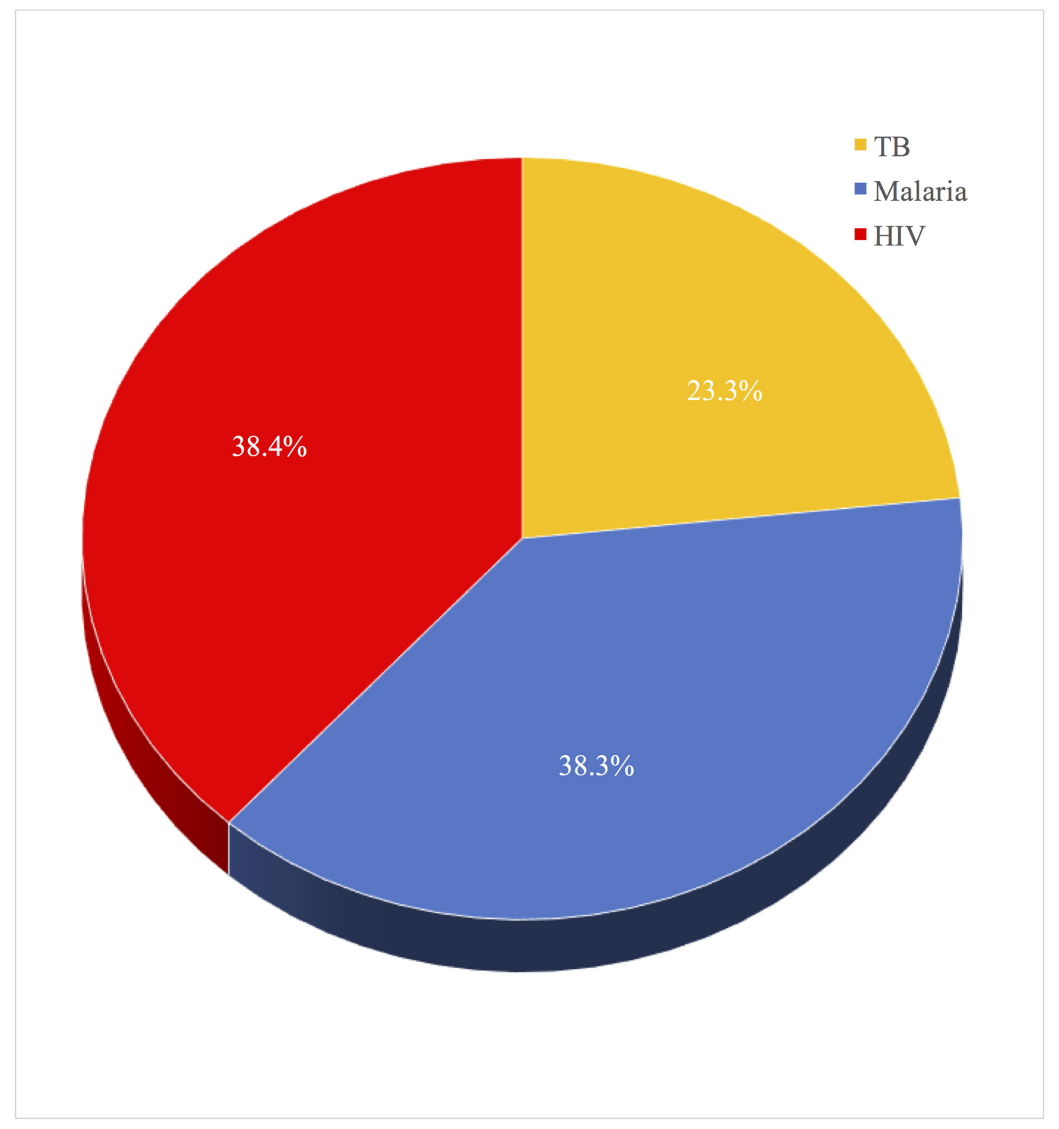
Total DALYS lost by disease. What proportion of DALYS lost due to all three diseases is attributable to malaria, TB, and HIV.

**Fig 3:**
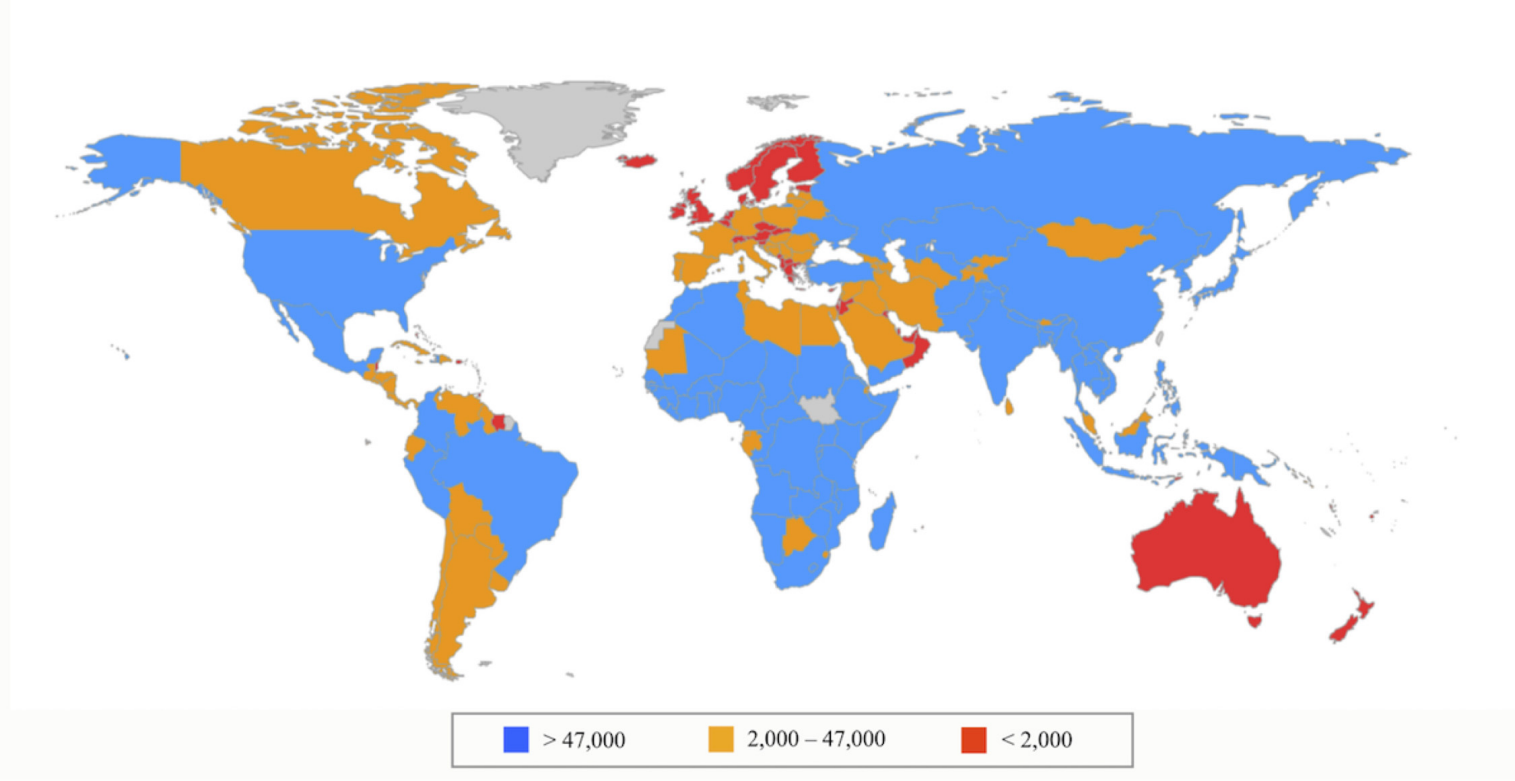
Estimated Disability Adjusted Life Years averted in each country.
