# Supplementary material for: The Global Health Impact Index: Promoting Global Health
Source: PLoS One. 2015 Dec 11;10(12):e0141374. doi: 10.1371/journal.pone.0141374 (PMC4676606; doi:10.1371/journal.pone.0141374)
Supplement: S1 Appendix — Contents: Section I. Drug Abbreviations. (p. 2) Table A, Drug Abbreviations. Section II. TB Example: How the Impacts of TB Drugs are Calculated. (p. 3) Fig A, Breakdown of TB patient groups. Table B, Impact on TB patient groups for Botswana in 2010. Table C, Disaggregated Drug-Susceptible TB Treatment Regimen. Table D, Possible MDR-TB Treatment Regimens. Table E, MDR-TB Resistance Patterns. Table F, Breakdown of TB Drug Resistance between Different Regimens. Table G, Portion of MDR-TB treatments. Table H, Proportion of Credit Given to Each Drug in Different Treatment Regimens. Table I, XDR-TB Treatment Regimen Drug Proportions. Table J, Disaggregated XDR-TB Treatment Regimens. Section III. HIV Example: How the Impacts of HIV Drugs are Calculated. (p. 11) Table K, Group A and Group B Countries Affected by HIV. Table L, Percentage of People Taking First and Second Line Regimens in Group A Countries. Table M, Antiretroviral Treatment Regimen Proportions and Efficacies for Group A and Group B Countries. Some of the material in this section is reprinted from “Globalization, Global Justice, and Global Health Impact” under a CC BY license, with permission from Public Affairs Quarterly, original copyright 2014 Section IV. Sensitivity Analyses. (p. 18) Fig B, Initial and Secondary Stability for Sensitivity Analyses. Section V. Drug Accreditation between Companies. (p. 25) Table N, Drug Accreditation between Companies. (DOCX) [file pone.0141374.s001.docx]

**Supporting Information**

**S1 Appendix. Drug Impacts, Sensitivity Analyses, and Company Accreditation.**

**Contents:**

**Section I. Drug Abbreviations. (p. 2)** Table A, Drug Abbreviations.

**Section II. TB Example: How the Impacts of TB Drugs are Calculated. (p. 3)**

Fig A, Breakdown of TB patient groups. Table B, Impact on TB patient groups for Botswana in 2010. Table C, Disaggregated Drug-Susceptible TB Treatment Regimen. Table D, Possible MDR-TB Treatment Regimens. Table E, MDR-TB Resistance Patterns. Table F, Breakdown of TB Drug Resistance between Different Regimens. Table G, Portion of MDR-TB treatments. Table H, Proportion of Credit Given to Each Drug in Different Treatment Regimens. Table I, XDR-TB Treatment Regimen Drug Proportions. Table J, Disaggregated XDR-TB Treatment Regimens.

**Section III. HIV Example: How the Impacts of HIV Drugs are Calculated. (p. 11)**

Table K, Group A and Group B Countries Affected by HIV. Table L, Percentage of People Taking First and Second Line Regimens in Group A Countries. Table M, Antiretroviral Treatment Regimen Proportions and Efficacies for Group A and Group B Countries. Some of the material in this section is reprinted from “Globalization, Global Justice, and Global Health Impact” under a CC BY license, with permission from *Public Affairs Quarterly*, original copyright 2014

**Section IV. Sensitivity Analyses. (p. 18)**

Fig B, Initial and Secondary Stability for Sensitivity Analyses.

**Section V. Drug Accreditation between Companies. (p. 25)**

Table N, Drug Accreditation between Companies.

**Section I. Drug Abbreviations.**

**Table A. Drug Abbreviations.**

| TB Drug Abbreviation | Full Name |
| --- | --- |
| 3TC | Lamivudine |
| ABC | Abacavir |
| AL | Artemether-Lumefantrine |
| Amk | Amikacin |
| AS+AQ | Artesunate + Amodiaquine |
| AS+MQ | Artesunate + Mefloquine |
| AS+SP | Artesunate + Sulfadoxine-Pyrimethamine |
| ATV/r | Atazanavir/Ritonavir |
| AZT | Zidovudine |
| Cm | Capreomycin |
| Cs | Cycloserine |
| DHA-PPQ | Dihydroarteminisin-Piperaquine |
| Ddl | Didanosine |
| d4T | Stavudine |
| E (or EMB) | Ethambutol |
| EFV | Efavirenz |
| Eto | Ethionamide |
| FTC | Emtricitabine |
| Gfx | Gatifloxacin |
| H (or INH) | Isoniazid |
| Km | Kanamycin |
| Lfx | Levofloxacin |
| LPV/r | Lopinavir with a ritonavir boost |
| Mfx | Moxifloxacin |
| NFV | Nelfinavir |
| NVP | Nevirapine |
| Ofx | Ofloxacin |
| PAS | 4-aminosalicylic acid |
| R (or RMP) | Rifampicin |
| S (or STR) | Streptomycin |
| TDF | Tenofovir |

**Section II. TB Example: How the Impacts of TB Drugs are Calculated.**

Consider how we calculate Pfizer’s drugs’ final impact score. Pfizer holds the original patent on four anti-TB drugs: ethambutol, pyrazinamide, cycloserine, and PAS. Ethambutol and pyrazinamide are used in combination with two other drugs (Isoniazid and Rifampicin) from other companies for treating (active) drug-susceptible TB. Cycloserine and PAS are also used in different combination therapies for multi-drug-resistant (MDR)-TB, and cycloserine is used in treating extremely drug-resistant (XDR)-TB as well [1].

The chart below outlines the breakdown of different patient groups we considered in crediting companies for anti-TB drugs.

**Figure A.** **Breakdown of TB patient groups.**


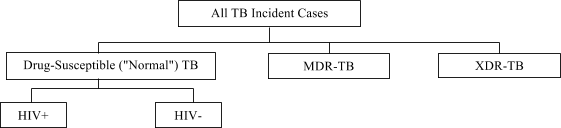


Given the above-mentioned basis of our model, the first step in calculating Pfizer’s impact score is as follows: Taking Botswana as an example country, we see that 18,661.80 DALYs were lost to TB in Botswana [2]. In 2010, the WHO reported 10,000 incident cases of TB in Botswana [3]. 80% of registered cases were tested for HIV status, and 65.43% of TB cases with known HIV status were HIV positive. So 8,000 (80% of 10,000) TB incident cases were tested for HIV, and the breakdown of HIV positive to HIV negative cases was 5,235 (65.43% of 8,000) to 2,765 (14.57% of 8,000). In countries where data is not available regarding the proportion of TB incident cases with known HIV status, an estimate was derived. This was done by using the global average of 33% of TB cases with a known HIV status [4]. Given that the global average is 33%, and given that we only have data on a proportion of the countries, we derived an estimate of the necessary average known HIV status for the remaining countries in order to reach the mean of 33%.

The next step involves breaking down incident cases into Drug-Susceptible TB, MDR-TB, and XDR-TB. We start with MDR-TB first. The WHO provides data for:

(a) Estimated numbers of MDR-TB cases among notified new cases of pulmonary TB: 120 in Botswana (Column e_new_mdr_num) [3].

(b) Estimated number of MDR-TB cases among notified previously treated pulmonary TB cases: 29 in Botswana (Column e_ret_mdr) [3].

(c) Estimated percentage of new TB cases with MDR-TB: 2.5% in Botswana (Column e_new_mdr_pcnt) [3].

(d) Estimated percentage of previously treated TB cases with MDR-TB: 7% in Botswana (Column e_ret_mdr_pcnt) [3].

Using this data, we determine:

(e) Estimated new cases (any type) = (a) / (c) = 120 / 2.5% = 4,800.00; and

(f) Estimated retreatment cases (any type) = (b) / (d) = 29 / 7% = 414.29

From this, we can calculate:

Overall percent MDR-TB among prevalent TB = [(a) + (b)] / [(e) + (f)]

= (120 + 29) / (4,800.00 + 414.29)

= 2.86%

This percentage is then multiplied by the prevalent cases: 2.86% * 8,100 (Column e_prev_num) [3]. = 231 MDR-TB cases needing treatment in Botswana in 2010.

The WHO provides data on the number of individuals treated per year. Based on 2010 WHO data, 92 individuals received MDR-TB treatment in Botswana (Column conf_mdr_tx) [3]. Our estimated treatment coverage, then, is the 92 individuals who received treatment divided by the 231 individuals needing treatment, or 39.75%.

To compute the DALYs lost to MDR-TB in Botswana, we use the same MDR-TB proportion of 2.86% of the total 18,661.80 DALYs due to TB of any type in Botswana to estimate that 533.27 DALYs were lost in 2010. From this, we then subtract the number of DALYs lost to XDR-TB cases (see below) to reach an estimate of actual DALYs lost to only MDR-TB of 485.27. Because treatment coverage for MDR-TB in Botswana is 39.75%, the impact of any MDR-TB regimen in Botswana, calculated by DALYs lost to MDR-TB * MDR-TB treatment coverage * efficacy of MDR-TB treatment, is 485.27% * 39.75% * 48%:^8^ 92.58 is the impact of MDR-TB treatment in Botswana.

For XDR-TB, we know that 9% of all MDR-TB cases are XDR-TB [5]. Multiplying this XDR-TB proportion to the total number of MDR-TB cases in Botswana, we have 9.00% out of 231 MDR-TB cases (or about 21 cases) being extensively drug-resistant. We have yet to obtain good data regarding country-level treatment coverage for XDR-TB [6]. Hence, we use global treatment coverage of 43% as an estimate [7]. Since 9.00% of MDR-TB cases are XDR-TB, we assume that this proportion is also representative of the DALYs lost to XDR-TB. Hence, we estimate that 9.00% of 533.27 DALYs lost to MDR-TB in Botswana in 2010, or 47.99 DALYs were lost to XDR-TB in Botswana. Efficacy of XDR-TB treatment is estimated at 20% [4]. Thus the impact of XDR-TB treatment in Botswana in 2010 is approximately 47.99 DALYs lost * 43% treatment coverage * 20% efficacy = 4.13.

Finally, we look at the treatment for Drug-Susceptible (or “Normal”) TB. As mentioned earlier, we assume that the DALYs lost to TB in general comes from the DALYs lost to Drug-Susceptible TB, MDR-TB, and XDR-TB. Based on this assumption, the DALYs lost to Drug Susceptible TB for Botswana in 2010 comes to 18,128.53. Previously, we calculated the number of HIV+ and HIV- cases among incident cases with known HIV status in Botswana were 5,235 and 2,765 respectively (of 8,000 cases in total). This works out to 65.43% HIV+ and 34.57% HIV-. Thus our DALY breakdown is as follows:

TB/HIV+: 18,128.53 * 65.43% = 11,861.73

TB/HIV-: 18,128.53 * 34.57% = 6,266.80

We have yet to get good treatment coverage data at the country level for each of the above cases. Thus for now we use the WHO’s estimate of the prevalence of directly observed treatment short-course (DOTS) coverage of 65.9% for all cases [8]. Estimated efficacy for TB/HIV+ treatment is 73% and that for TB/HIV- treatment is 87% [3]. Thus, impact scores for each case are calculated by DALYs lost * treatment coverage * treatment efficacy:

TB/HIV+: 11,861.73 * 65.9% * 73% = 5,706.32

TB/HIV-: 6,266.80 * 65.9% * 87% = 3,592.94

The following table provides a quick summary for all the scores we have calculated thus far for each scenario for Botswana in 2010:

**Table B. Impact on TB patient group**s **for Botswana in 2010.**

| TB Case | | Impact Score |
| --- | --- | --- |
| Drug-Susceptible (“Normal”) TB | TB/HIV+ | 5,706.32 |
|  | TB/HIV- | 3,592.94 |
| Multidrug-Resistant TB (MDR-TB) | | 92.58 |
| Extensively Drug-Resistant TB (XDR-TB) | | 4.13 |

The next step is to disaggregate these scores into the corresponding drugs that are involved in the treatment of Drug-Susceptible TB, MDR-TB, and XDR-TB.

**Table C. Disaggregated Drug-Susceptible TB Treatment Regimen.**

| Standard 6-month first-line regiment (2HRZE/4HR) | Drug Proportion of Regimen |
| --- | --- |
| Rifampicin | 0.25 |
| Isoniazid | 0.25 |
| Ethambutol | 0.25 |
| Pyrazinamide | 0.25 |

Again, we assume the impact of each drug in the standard 6-month regimen for active TB is equal.

**MDR-TB Treatment Regimens**

A total of three MDR-TB regimens are considered in this model. As explained in the paper, we estimate the percentage of people with MDR-TB receiving each regimen using data based on protocols and resistance rates [9, 10]. Once diagnostic susceptibility testing (DST) has been performed, and MDR-TB is confirmed, there are multiple possible regimens as indicated in the table below:

**Table D. Possible MDR-TB Treatment Regimens.**

|  | **Drug Resistance** | **Treatment Regimen** |
| --- | --- | --- |
| 1 | Resistant to isoniazid (H) in combination with rifampicin (R) or rifampicin and ethambutol (E) | Z + S + Lfx + Eto + Cs + PAS |
| 2 | Resistance to H+R+E+ pyrazinamide (Z) | S + Lfx + Eto + Cs + PAS |
| 3 | Resistance to any of the following: H+R+ streptomycin (S); H+R+E+S; or H+R+E+Z+S | Km + Lfx + Eto + Cs + PAS |

According to the National Center for Biotechnical Information, 39.4% of recipients of drug susceptibility tests indicate multiple drug resistance. The resistance patterns are indicated in the following table [10].

**Table E. MDR-TB Resistance Patterns.**

| **Drug Resistance** | **Estimated Proportion of Total MDR Cases (of those Receiving DST)** |
| --- | --- |
| Multidrug Resistance (total) | 39.4% |
| H+R | 7.1% |
| H+R+E | 3.3% |
| H+R+S | 11.0% |
| H+R+E+S | 18.6% |

Unfortunately, this data does not include pyrazinamide. Pyrazinamide resistance is difficult to test for, so many laboratories do not test for it [11]. Studies in South Africa, however, indicate resistance to pyrazinamide (Z) among MDR-TB cases of 42.25% (Table 2: 30/71) [11]. Given that H+R+E+Z+S is a subset of the H+R+E+S resistance in the second table above, then, we use the figure from South Africa to estimate that resistance to H+R+E+Z+S = 18.6% * 42.25% = 7.86%. We estimate that the remainder, 10.74% (18.6% - 7.86%) of the population, is resistant to H+R+E+S but *not* Z. Similarly, we multiply this percentage of 42.25% of those resistant to Z by the percentage of those resistant to H+R+E (3.3%) to get 1.39% resistant to H+R+E+Z and estimate that the remaining 1.91% are resistant to H+R+E, but *not* Z. We calculate the “proportion of MDR-TB” as the percentage of total TB divided by percentage of total TB that is MDR-TB (39.4%). Therefore, we estimate that resistances and the corresponding treatment regimens are as follows:

**Table F. Breakdown of TB Drug Resistance between Different Regimens.**

|  | **Drug Resistance** | **Portion of Total** | **Portion of Total MDR-TB** | **Treatment Regimen** | **Portion of MDR-TB treatment** |
| --- | --- | --- | --- | --- | --- |
| 1 | H+R | 7.1% | 18.02% | Z + S + Lfx + Eto + Cs + PAS | 22.86% |
|  | H+R+E, without Z | 1.91% | 4.84% |  |  |
| 2 | H+R+E+Z | 1.39% | 3.54% | S + Lfx + Eto + Cs + PAS | 3.54% |
| 3 | H+R+S | 11.0% | 27.92% | Km + Lfx + Eto + Cs + PAS | 75.13% |
|  | H+R+E+S without Z | 10.74% | 27.26% |  |  |
|  | H+R+E+Z+S | 7.86% | 19.95% |  |  |

Adjusted to 100%, the regimens proportions of MDR-TB treatment are as follows:

**Table G. Portion of MDR-TB treatments.**

|  | **Treatment Regimen** | **Portion of MDR-TB treatment** |
| --- | --- | --- |
| 1 | Z + S + Lfx + Eto + Cs + PAS | 22.51% |
| 2 | S + Lfx + Eto + Cs + PAS | 3.49% |
| 3 | Km + Lfx + Eto + Cs + PAS | 74.00% |

Within each regimen, we the give each drug equal credit. The proportion of credit given to each drug in each of the three regimens is shown in the right column in the table below.

**Table H. Proportion of Credit Given to Each Drug in Different Treatment Regimens.**

| Pyrazinamide + Streptomycin + Levofloxacin + Ethionamide + Cycloserine + PAS | Drug Proportion of Regimen |
| --- | --- |
| Pyrazinamide | 0.17 |
| Streptomycin | 0.17 |
| Levofloxacin | 0.17 |
| Ethionamide | 0.17 |
| Cycloserine | 0.17 |
| PAS | 0.17 |
|  |  |
| Streptomycin + Levofloxacin + Ethionamide + Cycloserine + PAS | Drug Proportion of Regimen |
| Streptomycin | 0.20 |
| Levofloxacin | 0.20 |
| Ethionamide | 0.20 |
| Cycloserine | 0.20 |
| PAS | 0.20 |
|  |  |
| Kanamycin + Levofloxacin + Ethionamide + Cycloserine + PAS | Drug Proportion of Regimen |
| Kanamycin | 0.20 |
| Levofloxacin | 0.20 |
| Ethionamide | 0.20 |
| Cycloserine | 0.20 |
| PAS | 0.20 |

**XDR-TB Treatment Regimen**

The treatment regimen considered for XDR-TB consists of cycloserine, at least one injectable second-line agent, and one fluoroquinolone [12]. Injectable second-line agents are kanamycin or amikacin or capreomycin and fluoroquinolones are levofloxacin or moxifloxacin or gatifloxacin or ofloxacin.

**Table I. XDR-TB Treatment Regimen Drug Proportions.**

| Regimen: Cycloserine + (Kanamycin or Amikacin or Capreomycin) + (Levofloxacin or Moxifloxacin or Gatifloxacin or Ofloxacin) | Drug Proportion of Regimen |
| --- | --- |
| Cycloserine | 0.33 |
| Kanamycin or Amikacin or Capreomycin | 0.11 (0.33/3) |
| Levofloxacin or Moxifloxacin or Gatifloxacin or Ofloxacin | 0.08 (0.33/4) |

Again, we give proportionate weight to each drug in the above XDR-TB regimen.

For Botswana, we disaggregate the scores as follows:

**Table J. Disaggregated XDR-TB Treatment Regimens.**

| TB Case | | Total Score times Proportion Receiving Relevant Treatment | Score Per Drug |
| --- | --- | --- | --- |
| Drug-Susceptible  ("Normal") TB | TB / HIV+ | 5,706.32 | Rifampicin: 0.25 * 5,706.32 = 1,426.58  Isoniazid: 0.25 * 5,706.32 = 1,426.58  Ethambutol: 0.25 * 5,706.32 = 1,426.58  Pyrazinamide: 0.25 * 5,706.32 = 1,426.58 |
|  | TB / HIV- | 3,592.94 | Rifampicin: 0.25 * 3,592.94 = 898.24  Isoniazid: 0.25 * 3,592.94 = 898.24  Ethambutol: 0.25 * 3,592.94 = 898.24  Pyrazinamide: 0.25 * 3,592.94 = 898.24 |
| Multidrug-Resistant TB (MDR-TB) | Resistance to isoniazid in combination with rifampicin or rifampicin and ethambutol | 20.84 | Pyrazinamide: 0.17 * 20.84 = 3.54  Streptomycin: 0.17 * 20.84 = 3.54 Levofloxacin: 0.17 * 20.84 = 3.54  Ethionamide: 0.17 * 20.84 = 3.54  Cycloserine: 0.17 * 20.84 = 3.54  PAS: 0.17 * 20.84 = 3.54 |
|  | Resistance to isoniazid with rifampicin, ethamutol, and pyrazinamide | 3.23 | Streptomycin: 0.20 * 3.23 = 0.65  Levofloxacin: 0.20 * 3.23 = 0.65  Ethionamide: 0.20 * 3.23 = 0.65  Cycloserine: 0.20 * 3.23 = 0.65  PAS: 0.20 * 8.48 = 3.23 = 0.65 |
|  | Resistance to isoniazid with rifampicin and streptomycin; isoniazid with rifampicin, ethamutol, and streptomcin; or resistance to isoniazid, rifampicin, ethambutol, pyrazinamide, and streptomycin | 68.51 | Kanamycin: 0.20 * 68.51 = 13.70  Levofloxacin: 0.20 * 68.51 = 13.70  Ethionamide: 0.20 * 68.51 = 13.70  Cycloserine: 0.20 * 68.51 = 13.70  PAS: 0.20 * 68.51 = 13.70 |
| Extensively Drug-Resistant TB (XDR-TB) | | 4.13 | Cycloserine: 0.33 * 4.13 = 1.36  Kanamycin: 0.11 * 4.13 = 0.45  Amikacin: 0.11 * 4.13 = 0.45  Capreomycin: 0.11 * 4.13 = 0.45  Levofloxacin: 0.08 * 4.13 = 0.33  Moxifloxacin: 0.08 * 4.13 = 0.33  Gatifloxacin: 0.08 * 4.13 = 0.33  Ofloxacin: 0.08 * 4.13 = 0.33 |

An individual drug’s score, then, is the sum of each of the proportional score of any regimen in which it is a part. Again, since this example is for Pfizer’s drugs’ impact score, we only focus on the drugs with patents held by this company: ethambutol, pyrazinamide, cycloserine and PAS. The impact score for ethambutol, pyrazinamide, cycloserine, and PAS in Botswana is simply the sum of individual scores in the table above that are associated with each drug respectively. The total score for Pfizer is the summation across all countries in the model, which sums up to 13,209,904.58 in all countries in the world.

**Section III. HIV Example: How the Impacts of HIV Drugs are Calculated.**

Consider how we calculate Shire Pharmaceutical’s drug’s score. Shire Pharmaceutical holds the original patent on only one antiretroviral drugs for HIV: Lamivudine (3TC). Here we do not consider different disease states, and set aside questions about interactions between HIV drugs and others for a rough estimate of impact.

The HIV scoring model is based on WHO data collected from mid- and low-income countries affected by HIV that responded to the WHO AIDS Medicines and Diagnostics Service (AMDS) survey. These countries were classified by the WHO as either "Group A" or "Group B" countries. The following table shows the list of countries that responded to the WHO AIDS Medicine and Diagnostic Service survey [13].

**Table K. Group A and Group B Countries Affected by HIV.**

| **Group A**  *Low- and Middle-Income Countries excluding region of the Americas* | | **Group B**  *Low- and Middle-Income Countries in the Americas* |
| --- | --- | --- |
| Afghanistan  Bangladesh  Belarus  Bhutan  Botswana  Burkina Faso  Burundi  Cambodia  Cameroon  Central African Republic  China  Democratic Republic of the Congo  Gambia  Ghana  Guatemala  India  Iran  Kenya  Lesotho  Madagascar  Malawi  Malaysia  Mozambique | Myanmar  Namibia  Nepal  Oman  Papua New Guinea  Qatar  Republic of Moldova  Romania  Saudi Arabia  Sierra Leone  Somalia  Sri Lanka  Sudan  Suriname  Swaziland  Tanzania  Uganda  Ukraine  United Arab Emirates  Viet Nam  Yemen  Zambia  Zimbabwe | Anguilla  Antigua and Barbuda  Argentina  Belize  Bolivia  Brazil  Chile  Cuba  Dominican Republic  Ecuador  El Salvador  Grenada  Guyana  Honduras  Nicaragua  Panama  Paraguay  Peru  Trinidad and Tobago  Uruguay |

Again the general formula for calculating the impact score for any drug is DALYs * % Treatment Coverage * Drug Efficacy. Because the WHO presents statistics for adults (defined as 15 years of age and above) and children (defined as below 15 years of age) separately, the model starts by calculating impact for these patient groups. Conveniently, the Global Health Data Exchange provides such age-specific DALYs information [14]. Using Somalia as an example, 215,359.79 DALYs were lost by adults to HIV in 2010, and 50,428.55 were lost by children [15].

The WHO provides data for numbers of individuals (all age groups) needing treatment and number of individuals (all age groups) receiving treatment [16]. Additionally, the same data is provided for children (<15) needing treatment and children receiving treatment [16]. From these numbers, the number of adults needing and receiving treatment can be easily calculated. In Somalia, for example, 25,000 people (all age groups) need treatment, 6,300 of which are children. The number of adults needing treatment, then, is the remaining 18,700. Similarly, 878 people are receiving treatment, 34 of whom are children; the remaining 844, then, are adults. Treatment coverage in Somalia, then, is 4.51% for adults (844/18,700) and 0.54% for children (34/6300). If country-specific treatment numbers are not provided, then numbers are calculated based on what would be necessary to reach regional averages [17, 18].

Recall that the WHO provides information about what percentage of adults and children are taking first and second line regimens by country group in Group A and B countries. Here is the information for Group A countries [19].

**Table L. Percentage of People Taking First and Second Line Regimens in Group A Countries.**

| **ADULTS** | **Group A** |
| --- | --- |
| First-Line Regimens | 97.10% |
| Second-Line Regimens | 2.90% |
|  |  |
| **CHILDREN** | **Group A** |
| First-Line Regimens | 96.80% |
| Second-Line Regimens | 3.20% |

Again, we also assume that the DALYs each regimen can recover are proportionate to their use in each population.

The next table lists the first and second line antiretroviral regimens and efficacy information for adults and children in each group [19].

**Table M. Antiretroviral Treatment Regimen Proportions and Efficacies for Group A and Group B Countries** [13]**.**

**Group A**

| ADULT First-Line Regimens | Proportion of Adult First- Line Regimens | Efficacy (%) |
| --- | --- | --- |
| Stavudine + Lamivudine + Nevirapine  Zidovudine + Lamivudine + Nevirapine  Stavudine + Lamivudine + Efavirenz  Zidovudine + Lamivudine + Efavirenz  Tenofovir + Lamivudine + Efavirenz  Tenofovir + Emtricitabine +Efavirenz  Tenofovir + Lamivudine + Nevirapine  Tenofovir + Emtricitabine + Nevirapine  Others | 27.70%  26.80%  14.00%  11.40%  10.60%  3.50%  2.70%  2.50% 0.80% | 69.65%  77.00%  77.50%  72.03%  76.73%  81.06%  75.00%  76.70%  69.65% |
| ADULT Second-Line Regimens | Proportion of Adult Second-Line Regimens | Efficacy (%) |
| Tenofovir + Lamivudine + Lopinavir/Ritonavir  Zidovudine + Didanosine + Lopinavir/Ritonavir  Zidovudine + Lamivudine + Lopinavir/Ritonavir  Tenofovir + Emtricitabine + Lopinavir/Ritonavir  Zidovudine + Lamivudine + Tenofovir + Lopinavir/Ritonavir  Abacavir + Didanosine + Lopinavir/Ritonavir  Abacavir + Tenofovir + Lopinavir/Ritonavir  Stavudine + Lamivudine + Lopinavir/Ritonavir  Abacavir + Lamivudine + Lopinavir/Ritonavir  Others | 27.10%  25.00%  12.70%  10.70%  5.50%  4.80%  2.50%  1.90  1.10%  8.70% | 83.00%  64.74%  50.00%  67.00%  64.74%  64.74%  64.74%  60.50%  63.20%  64.74% |
| **CHILDREN First-Line Regiments** | **Proportion of Children First-Line Regimens** | **Efficacy (%)** |
| Stavudine + Lamivudine + Nevirapine  Zidovudine + Lamivudine + Nevirapine  Stavudine + Lamuviudine + Efavirenz  Zidovudine + Lamivudine + Efavirenz  Abacavir + Lamivudine + Efavirenz  Stavudine + Lamivudine + Lopinavir/Ritonavir  Abacavir + Lamivudine + Lopinavir/Ritonavir  Abacavir + Lamivudine + Nevirapine  Others | 34.90%  20.70%  15.60%  7.20%  6.20%  5.90% 5.80%  1.70%  1.50% | 100.00%  78.50%  77.50%  72.03%  59.00%  62.00%  63.20%  73.18%  73.18% |
| **CHILDREN Second-Line Regiments** | **Proportion of Children Second-Line Regimens** | **Efficacy (%)** |
| Abacavir + Lamivudine + Lopinavir/Ritonavir  Zidovudine + Didanosine + Lopinavir/Ritonavir  Abacavir + Didanosine + Lopinavir/Ritonavir  Zidovudine +Lamivudine + Lopinavir/Ritonavir  Zidovudine + Didanosine + Efavirenz  Stavudine + Lamivudine + Lopinavir/Ritonavir  Tenofovir + Lamivudine + Lopinavir/Ritonavir  Zidovudine + Abacavir + Lamivudine + Lopinavir/Ritonavir  Stavudine + Lamivudine + Abacavir  Others | 26.20%  17.20%  14.80%  12.30%  6.60%  4.60%  2.00%  1.60%  1.40%  13.30% | 63.20%  65.34%  65.34%  50.00%  65.34%  60.50%  83.00%  70.00%  65.34%  65.34% |

**Group B**

| ADULT First-Line Regimens | Proportion of Adult First- Line Regimens | Efficacy (%) |
| --- | --- | --- |
| Zidovudine + Lamivudine + Efavirenz  Zidovudine +Lamivudine + Lopinavir/Ritonavir  Zidovudine + Lamivudine + Nevirapine  Zidovudine + Lamivudine + Atazanavir/Ritonavir  Tenofovir + Emitricitabine + Efavirenz  Abacavir + Lamivudine + Efavirenz  Stavudine + Lamivudine + Nevirapine  Stavudine + Lamivudine + Efavirenz  Others | 42.50%  13.60%  12.00%  6.40%  6.20%  2.60%  2.10%  1.80%  12.90% | 82.00%  50.00%  65.33%  72.78%  83.87%  71.00%  79.77%  77.50%  72.78% |
| ADULT Second-Line Regimens | Proportion of Adult Second-Line Regimens | Efficacy (%) |
| Tenofovir + Lamivudine + Efavirenz  Tenofovir + Lamivudine + Lopinavir/Ritonavir  Tenofovir + Lamivudine + Atazanavir/Ritonavir  Zidovudine + Lamivudine + Lopinavir/Ritonavir  Zidovudine + Lamivudine + Tenofovir + Lopinavir/Ritonavir  Stavudine + Lamivudine + Efavirenz  Stavudine + Lamivudine + Lopinavir/Ritonavir  Tenofovir + Lamivudine + Nevirapine  Zidovudine + Lamivudine + Tenofovir + Atazanavir/Ritonavir  Others | 18.10%  16.60%  13.40%  3.90%  3.00%  2.60%  2.20%  1.70%  1.40%  37.00% | 76.73%  83.00%  77.00%  50.00%  68.75%  77.50%  59.00%  58.00%  68.75%  68.75% |
| **CHILDREN First-Line Regiments** | **Proportion of Children First-Line Regimens** | **Efficacy (%)** |
| Zidovudine + Lamivudine + Efavirenz  Zidovudine + Lamivudine + Lopinavir/Ritonavir  Zidovudine + Lamivudine + Nevirapine  Zidovudine + Lamivudine + Nelfinavir  Zidovudine + Didanosine + Lopinavir/Ritonavir  Zidovudine + Didanosine + Efavirenz  Others | 32.10%  26.70%  17.50%  3.50%  3.30%  2.60%  14.40% | 72.35%  50.00%  78.50%  66.95%  66.95%  66.95%  66.95% |
| **CHILDREN Second-Line Regiments** | **Proportion of Children Second-Line Regimens** | **Efficacy (%)** |
| Zidovudine + Lamivudine + Nevirapine  Zidovudine +Lamivudine + Lopinavir/Ritonavir  Zidovudine + Lamivudine + Nevirapine  Zidovudine + Lamivudine + Nelfinavir  Zidovudine + Lamivudine + Lopinavir/Ritonavir  Zidovudine + Lamivudine + Efavirenz  Others | 32.10%  26.70%  17.50%  3.50%  3.30%  2.60%  14.40% | 72.03%  50.00%  78.50%  66.84%  66.84%  66.84%  66.84% |

Finally, remember that in each of these drug regimens, we give equal weight to each of the drugs that make up the regimen. In first-line adult regimens in A countries, for example, efavirenz is credited with 1/3 of the proportion credited to the regimen tenofovir + lamivudine + efavirenz: (1/3) * 10.60% = 3.53%.

Let us look, then, at how lamivudine’s score is calculated in Somalia. In Somalia 215,359.79 DALYs were lost in the adult sub-population and 50,428.55 in the child-sub-population in 2010 [2]. Somalia is classified as an “A” country by the WHO, so its treatment proportions and the efficacies of specific regimens are represented by the “Group A” section of the chart above. Using our basic formula of Need * Efficacy * Coverage, we can calculate lamivudine’s impact in the various regimens of which they are a part.

We will begin by calculating the impact of lamivudine in adult first-line treatment regimens. The first adult first-line treatment regimen containing lamivudine is “Stavudine + Lamivudine + Nevirapine” in the chart above. Recall that adult DALYs in Somalia amount to 215,359.79, and treatment coverage for adults in Somalia was calculated to be 4.51% (see above). These are the “Need” and “Treatment Percentage” factors in our equation. We then multiply this by the percent of adults that receive first-line treatment (97.1%), the proportion of those adult first-line treatments that receive Stavudine + Lamivudine + Nevirapine (27.70%), and the efficacy of that treatment (69.65%). Then, since Lamivudine is one of three drugs in the drug regimen, it receives 1/3 of this impact score: 215,359.79 * 4.51 % * 97.1% * 27.70% * 69.65% * (1/3) = 24.52. This is then repeated for each regimen of which lamivudine is a part in adult first-line treatment regimens in WHO-classified “A” countries since Somalia is an A country:

- Stavudine + Lamivudine + Nevirapine:

215,359.79 * 4.51 % * 97.1% * 27.70% * 69.65% * (1/3) = 606.51

- Zidovudine + Lamivudine + Nevirapine:

215,359.79 * 4.51 % * 97.1% * 26.80% * 77.00% * (1/3) = 648.73

- Stavudine + Lamivudine + Efavirenz:

215,359.79 * 4.51 % * 97.1% * 14.00% * 77.50% * (1/3) = 341.09

- Zidovudine + Lamivudine + Efavirenz:
- 215,359.79 * 4.51 % * 97.1% * 11.40% * 72.03% * (1/3) = 258.14
- Tenofovir + Lamivudine + Efavirenz:

215,359.79 * 4.51 % * 97.1% * 10.60% * 76.73% * (1/3) = 255.69

- Tenofovir + Lamivudine + Nevirapine:

215,359.79 * 4.51 % * 97.1% * 2.70% * 75.00% * (1/3) = 63.66

And adult second-line treatment regimens in WHO-classified “A” countries that contain lamivudine are:

- Tenofovir + Lamivudine + Lopinavir/Ritonavir:

215,359.79 * 4.51 % * 2.90% * 27.10% * 83.00% * (1/3) = 21.12

- Zidovudine + Lamivudine + Lopinavir/Ritonavir:

215,359.79 * 4.51 % * 2.90% * 12.70% * 50.00% * (1/3) = 5.96

- Zidovudine + Lamivudine + Tenofovir + Lopinavir/Ritonavir:

215,359.79 * 4.51 % * 2.90% * 5.50% * 64.74% * (1/4) = 2.51

- Stavudine + Lamivudine + Lopinavir/Ritonavir:

215,359.79 * 4.51 % * 2.90% * 1.90% * 60.50% * (1/3) = 1.08

- Abacavir + Lamivudine + Lopinavir/Ritonavir:

215,359.79 * 4.51 % * 2.90% * 1.10% * 63.20% * (1/3) = 0.65

And children first-line treatment regimens in WHO-classified “A” countries contain lamivudine are:

- Stavudine + Lamivudine + Nevirapine:

50,428.55 * 0.54% * 96.80%* 34.90% * 100.00% * (1/3) = 30.67

- Zidovudine + Lamivudine + Nevirapine:

50,428.55 * 0.54% * 96.80%* 20.70% * 78.50% * (1/3) = 14.28

- Stavudine + Lamuviudine + Efavirenz:

50,428.55 * 0.54% * 96.80%* 15.60% * 77.50% * (1/3) = 10.62

- Zidovudine + Lamivudine + Efavirenz:

50,428.55 * 0.54% * 96.80%* 7.20% * 72.03% * (1/3) = 4.56

- Abacavir + Lamivudine + Efavirenz:

50,428.55 * 0.54% * 96.80%* 6.20% * 59.00% * (1/3) = 3.21

- Stavudine + Lamivudine + Lopinavir/Ritonavir:

50,428.55 * 0.54% * 96.80%* 5.90% * 62.00% * (1/3) = 3.21

- Abacavir + Lamivudine + Lopinavir/Ritonavir:

50,428.55 * 0.54% * 96.80%* 5.80% * 63.20% * (1/3) = 3.22

- Abacavir + Lamivudine + Nevirapine:
- 50,428.55 * 0.54% * 96.80%* 1.70% * 73.18% * (1/3) = 1.09

And children second-line treatment regimens in WHO-classified “A” countries contain lamivudine are:

- Abacavir + Lamivudine + Lopinavir/Ritonavir:

50,428.55 * 0.54% * 3.20%* 26.20% * 63.20% * (1/3) = 0.48

- Zidovudine +Lamivudine + Lopinavir/Ritonavir:

50,428.55 * 0.54% * 3.20%* 12.30% * 50.00% * (1/3) = 0.18

- Stavudine + Lamivudine + Lopinavir/Ritonavir:

50,428.55 * 0.54% * 3.20%* 4.60% * 60.50% * (1/3) = 0.08

- Tenofovir + Lamivudine + Lopinavir/Ritonavir:

50,428.55 * 0.54% * 3.20%* 2.00% * 83.00% * (1/3) = 0.05

- Zidovudine + Abacavir + Lamivudine + Lopinavir/Ritonavir:

50,428.55 * 0.54% * 3.20%* 1.60% * 70.00% * (1/4) = 0.02

- Stavudine + Lamivudine + Abacavir:

50,428.55 * 0.54% * 3.20%* 1.40% * 65.34% * (1/3) = 0.03

The overall impact for lamivudine in Somalia, then, is:

606.51 + 648.73 + 341.09 + 258.14 + 255.69 + 63.66 + 21.12 + 5.96 + 2.51 + 1.08 + 0.65 + 30.67 + 14.28 + 10.62 + 4.56 + 3.21 + 3.21 + 3.22 + 1.09 + 0.48 + 0.18 + 0.08 + 0.05 + 0.02 + 0.03 = approximately 2278.45

This is also the overall impact for Shire Pharmaceutical’s drugs in Somalia. When this is performed for every country, the overall impact of Shire Pharmaceutical’s drugs on HIV is 7,431,422.84.

**Section IV. Sensitivity Analyses.**

We report below the results of 27 sensitivity analyses to test the stability of the rating model to key assumptions and methods. Of these, 10 caused no change whatsoever in overall rank. An additional 10 saw only two degrees of change, wherein two companies’ positions flipped in ranked position. Only 7 saw more than these two degrees of change. We have not attempted to correct for all causes of potential bias in our different data sources, but do not believe that cumulatively these sources will bias our estimates in any particular direction. The results of each test follow.

**Test #2:**

**Assumption tested**: In the malaria calculations, average global efficacy of a drug is used as fallback data for that drug if efficacy data is not available for a country. Here, we test using maximum or minimum treatment efficacy for the relevant drugs instead of the average.

**Response**: Minimal changes in score were observed, but no change in overall ranking occurred when using either maximum or minimum treatment efficacy as fallback data points.

**Test #5:**

**Assumption tested**: We assumed the proportion of total TB DALYs lost to MDR-is equal to the proportion of total TB cases that are MDR-TB cases.

**Response**: The model is stable if we attribute up to 59% fewer DALYs to MDR-TB than the corresponding proportion of overall TB cases that are MDR-TB. Similarly, the model is stable with up to a 686% increase in DALYs attributable to MDR-TB than the proportion of overall TB cases. Outside of this range, two companies flip. An additional flip in company rankings is seen at a 72% decrease in the proportion of DALYs in relation to the proportion of MDR-TB cases.

**Test #6:**

**Assumption tested**: Sources indicated that MDR-TB treatment is 48% efficacious. We assume this applies to all countries.

**Response**: The model shows no change in overall rank for MDR-TB treatment efficacy between 13% and 100%. At 13%, there is a flip in company rankings.

**Test #8**:

**Assumption tested**: Global XDR-TB treatment is estimated at 43%. Here, we test the impact of greater or lesser XDR-TB treatment percentages.

**Response**: The model is stable with treatment percentages for XDR-TB between 0% and 100%.

**Test #9**:

**Assumption tested**: We assume that the proportion of DALYs attributable to XDR-TB is equal to the proportion of XDR-TB cases among MDR-TB cases. Here we test attributing a greater or lesser proportion of DALYs to XDR-TB than the proportion of XDR-TB cases.

**Response**: The model is stable with up to a 224% greater proportion of DALYs attributable to XDR-TB than the proportion of cases that are XDR-TB. Beyond that, a flip is seen in company rank. No decrease in the proportion of DALYs attributable to XDR-TB sees a change in ranking.

**Test #10**:

**Assumption tested**: XDR-TB treatment efficacy is estimated at 20%. Here, we test the impact of greater or lesser XDR-TB treatment efficacy.

**Response**: Model is stable with XDR-TB treatment efficacy between 0% and 65%. At 65%, a flip in company rank is observed. No further change in ranking is observed until 87%, at which point an additional four companies have observed changes in ranking.

**Test #12**:

**Assumption tested**: Treatment coverage for “normal” TB is estimated to be 65.9%. Here, we test the impact of increasing or decreasing this estimate of treatment coverage.

**Response**: Rankings are stable with estimated treatment coverage for “normal” TB between 44% and 68%. Outside of this range flips in ranking are observed between two companies. Additional flips in ranking are observed at 75% and 37%.

**Test #16**:

**Assumption tested**: For HIV, the proportion of DALYs recovered due to first-, second-, or third-line treatment is assumed to be equivalent to the proportion of treatments that are first-, second-, or third-line. Here we test extending a greater proportion of DALYs to either first-, or second- and third-line, treatments.

**Response**: Model is stable with the proportion DALYs being recovered due to second- and third-line treatment being up to 515% of the proportion of treatment that is second- and third-line. At 516%, a flip in ranking is observed between two companies. Reducing DALYs recovered due to 2nd- and 3rd-line treatment was stable until 34%, at which point a flip in ranking was observed.

**Test #17**:

**Assumption tested**: The WHO will occasionally indicate TB incidence in a given country of “<10”. When this is the case, we have estimated the incidence of TB within that country as 10 cases. Setting the estimated incidence of TB in these countries at 0.

**Response**: The test caused no change in ranking.

**Test #18**:

**Assumption tested**: For HIV, the number needing treatment—both overall (for adults and children) and for children only—is given as a range. We take the mean of the range. Here we test using either the upper or lower bound.

**Response**: No change in company ranking resulted.

**Test #19**:

**Assumption tested**: WHO data indicates some countries have no HIV+ patients among those with TB. Here we see what happens when we assume there are some cases in these countries.

**Response**: Assuming the average number of HIV+ cases in all countries with zero reported HIV+ caused no change in ranking.

**Test #20**:

**Assumption tested**: Treatment regimen for XDR-TB consists of: (1) Cycloserine, (2) an injectable 2nd-line agent, and (3) one fluoroquinolone. Each of these is given equal weight in estimating impact of the treatment regimen. Here, we test giving these three components of the regimen different weights.

**Response**: The model is stable from 0% to 100% of the weight given to cycloserine.

**Test #22**:

**Assumption tested**: Assumption that where ACT coverage exists within a country but no first-line drug is specified, each of the ACTs in our model could eliminate 1/11^th^ of the possible DALYS lost to p. Falciparum malaria in the county. Here, we test not giving any of the ACTs any credit in these countries.

**Response**: No change in ranking is observed.

**Test #25**:

**Assumption tested**: Currently, if efficacy for a particular drug regimen as applied to a specific subgroup of individuals (e.g. first-line adult treatment) is not available, we will use average efficacy for that drug among all subjects. If this data does not exist, we will use the average efficacy for that subgroup of individuals. Here, instead of first turning to average efficacy of the drug for all subjects, we turn to average efficacy for that subgroup.

**Response**: No change in ranking is observed.

**Test #26**:

**Assumption tested**: For malaria efficacy estimates we use data only from the 2010 World Malaria Report. In this test, we include missing data points from older reports.

**Response**: No change in ranking is observed.

**Test #28**:

**Assumption tested**: Proportion of DALYs that could be saved by a particular treatment regimen in a country when there are multiple malaria treatments within a country is currently divided by the number of malaria treatments. Here, we instead calculate the percent of countries wherein each treatment regimen is used. In a country with multiple drug regimens, each drug regimen is credited with the its percentage divided by the total of the percentages of all drugs present (e.g. if two drugs are used in a country, drug A and drug B, and drug A is used in 25% of countries and drug B in 50%, drug A is credited with treating 25% / (25% + 50%) of the DALYs).

**Response**: Six companies saw changes in ranking.

**Test #29**:

**Assumption tested**: The model extends information on HIV WHO group (“A” or “B”) to countries that are not classified as such by the WHO [15]. Extrapolated “A” and “B” status is decided by region. Here, we test excluding these countries when calculating either just HIV treatment impact or impact of treatment for all diseases (malaria, TB, HIV).

**Response**: Excluding countries with missing data in HIV only resulted in change in ranking for five different companies. If these countries were excluded from all disease models, changes in ranking occurred for eight companies.

**Test #31**:

**Assumption tested**: The model uses DALYs to calculate impact scores. Here, we test using mortality data instead.

**Response**: Ten companies see a change in ranking when mortality is used instead of DALYs.

**Test #33**:

**Assumption tested**: Impact scores are included for countries of all incomes. Here we test excluding high- and upper middle-income countries, either from HIV alone or from all disease types (malaria, TB, HIV).

**Response**: When excluding high- and upper-middle income countries from HIV, three companies had a change in ranking. The same three companies had a change in rank when excluding high- and upper-middle income countries from all disease impacts.

**Test #34**:

**Assumption tested**: In the model the amount of credit given to each MDR-TB treatment regimen is inversely proportional to the resistance to those drugs [10]. Here, we test giving each MDR-TB treatment regimen equal weight.

**Response**: No change in ranking observed when giving each MDR-TB treatment regimen equal weight instead of weighting each in inverse proportion to the resistance exhibited to that regimen.

**Test #38**:

**Assumption tested**: The four drugs in the standard TB regimen receive equal weight. Here, we test modifying the proportion given to isoniazid, and dividing the remainder equally between rifampicin, ethambutol, and pyrazinamide.

**Response**: Isoniazid normally receives 25% of the impact of the standard regimen. This is stable between 23% and 29%. Outside of those bounds, a flip in company ranking is observed.

**Test #39**:

**Assumption tested**: TB/HIV+ treatment efficacy is estimated at 73%. Here, we test the impact of increasing or decreasing the estimate of treatment efficacy.

**Response**: The model is stable with TB/HIV- treatment efficacy between 0% and 91%. At 91%, a flip in rank is observed.

**Test #40**:

**Assumption tested**: TB/HIV- treatment efficacy is estimated at 87%. Here, we test the impact of increasing or decreasing this estimate of treatment efficacy.

**Response**: The model is stable with estimated TB/HIV- treatment efficacy between 55% and 90%. Setting efficacy outside of this range saw a flip in ranking between two companies. No further flip is seen with efficacy of up to 100%, but a further flip is seen below 44%.

**Test #41**:

**Assumption tested**: Survey data is used for ACT treatment coverage for malaria. If country-specific survey data is not available, then the regional average of available data is used. If no data is available for the region, then the global average is used. Here, we perform three tests: (a) use country-specific survey data first for Malaria treatment efficacy, then global average survey data as fallback (rather than region-specific); (b) use country-specific survey data first, then WHO data if available, then average of all countries where those two data points are available; and (c) use only WHO data--country-specific where available, then average where not.

**Response**: Each of these tests resulted in one flip in company ranking.

**Figure B. Initial and Secondary Stability for Sensitivity Analyses.**


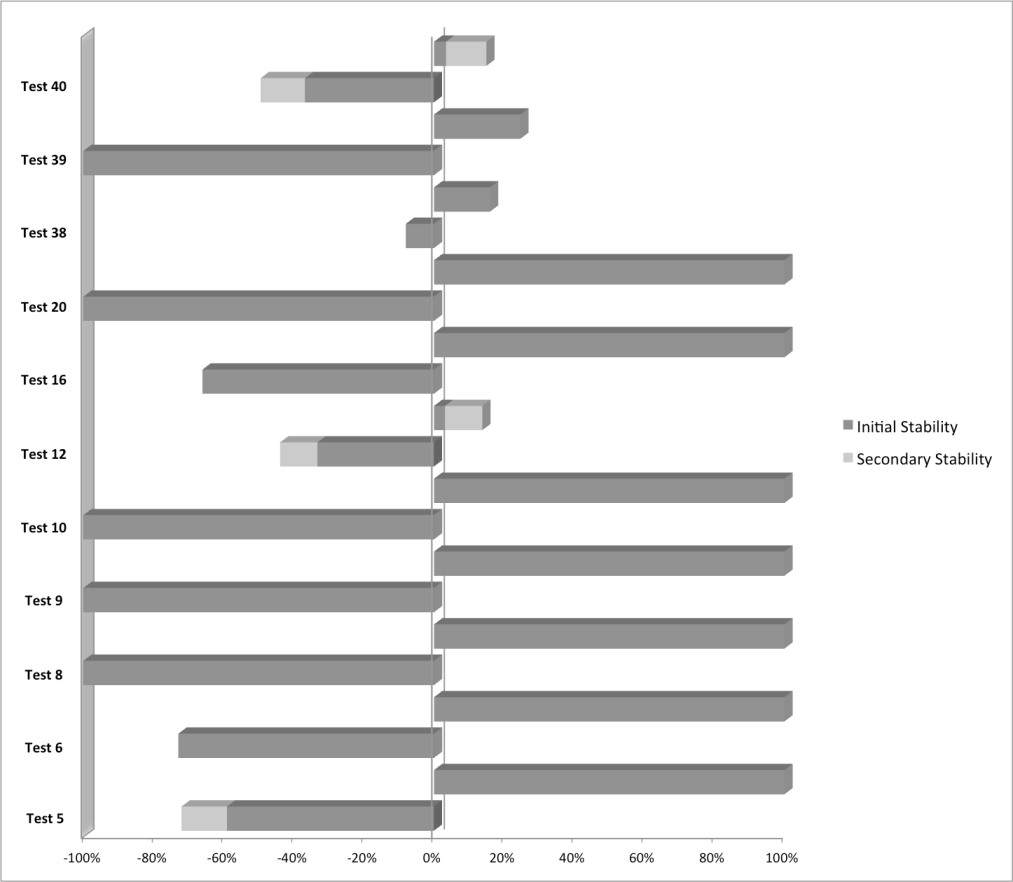


**Section V.** **Drug Accreditation between Companies.**

**Table N. Drug Accreditation between Companies.**

| Disease | Drug | Abbreviation | Company | Reference |
| --- | --- | --- | --- | --- |
| HIV | Abacavir | ABC | ViiV Healthcare/ GlaxoSmithKline | Abacavir was formerly known as 1492u89 [20]. The first patent for 1492u89 was by The Wellcome Foundation Limited [21]. The Wellcome Foundation Limited was merged with Glaxo in 1995 to form Glaxo Wellcome [22]. Glaxo Wellcome then merged with SmithKline Beecham in January 2000 to form GlaxoSmithKline [23]. ViiV was created in 2009 as a joint venture between GlaxoSmithKline and Pfizer to take over their HIV operations [24]. |
|  | Atazanavir/Ritonavir | ATV/r | Novartis; Abbott Pharmaceuticals | The earliest patent for Atazanavir, an antiretroviral drug used in HIV/AIDS treatment, was by Novartis in 1995 (Ciba-Geigy, at the time) [25]. Ritonavir was first patented by Abbott Laboratories in 1993 [26]. |
|  | Didanosine | ddl | Bristol Myers Squibb | Didanosine was developed in the National Cancer Institute (NCI) by Samuel Broder, Hiroaki Mitsuya, and Robert. Given that the NCI cannot market a product, Bristol-Myers Squibb was awarded a ten-yaer exclusive license to market and cell ddl as Videx® tablets by the National Institute of Health [27]. |
|  | Efavirenz | EFV | Merck | First patented by Merck in 1996 [28]. |
|  | Emtricitabine | FTC | Gilead Sciences, Inc. | Emtricitabine was first developed by scientists at Emory University [29]. Gilead subsequently paid $525 million for the royalties due to Emory for the drug [30]. |
|  | Lamivudine | 3TC | Shire Pharmaceuticals | IAF Biochem first patented Lamivudine in Patent Number 5047407 [31]. IAF subsequently changed its name to Biochem Pharma, which was then merged with Shire Pharmaceuticals in 2000 [32]. |
|  | Lopinavir with a ritonavir boost | LPV/r | Abbot Laboratories | Patent Number 5541206 [33]. |
|  | Nelfinavir | NFV | ViiV Healthcare | Nelfinavir was first developed by the Agouron Institute [34]. Agouron was sold to Warner Lambert in 1998, which subsequently merged with Pfizer [35]. ViiV was created in 2009 as a joint venture between GlaxoSmithKline and Pfizer to take over their HIV operations (see above reference, Abacavir). |
|  | Nevirapine | NVP | Boehringer Ingelheim | Patent Number EP 0667348 [36]. |
|  | Stavudine | d4T | Bristol Myers Squibb | Patent Number 5539099 [37]. |
|  | Tenofovir | TDF | Gilead Sciences, Inc. | Tenofovir was patented by Gilead Sciences, Inc. in 1998 (filed in 1996) [38]. |
|  | Zidovudine | AZT | ViiV | Glaxo filed the first patent for zidovudine in 1992 [39]. Through a series of mergers, Glaxo is now GlaxoSmithKline (see abacavir, above). ViiV was created in 2009 as a joint venture between GlaxoSmithKline and Pfizer to take over their HIV operations (see above reference, Abacavir). |
| TB | Amikacin | Amk | Bristol Myers Squibb | Patent Number 4206116 (about the combination of amikacin and penicillin) references amikacin as “those pharmaceutically acceptable acid addition salts disclosed in U.S. Pat. No. 3,781,268 as being included within the scope of the invention claimed therein” [40]. Patent 3,781,268 was issued to Bristol Myers Squibb in 1973 [41]. |
|  | Capreomycin | Cm | Eli Lilly | Capreomycin was originally isolated by Eli Lilly & Co. in 1961 [42]. |
|  | Cycloserine | Cs | Pfizer, Inc. | Patent Gilbert M. Shull *et al*. first applied for a patent for Cycloserine and the production thereof in 1952 for Pfizer [43]. |
|  | Ethambutol | E | Pfizer, Inc. | The earliest patent for Ethambutol was by the American Cyanamid Company, filed on August 1, 1974 and issued March 16, 1976 [44]. American became a subsidiary of American Home Products Corp. in 1995 [45]. American Home Products eventually changed its name to Wyeth [46], and Wyeth was subsequently acquired by Pfizer [47]. |
|  | Ethionamide | Eto | Sanofi | The earliest patent for Ethionamide dates to 1959 (filed 1957) by Chimie et Atomistique [48]. The last patent that belongs to Chimie et Atomistique is from 1962 [49]. However, it seems that Francois Albert created the pharmaceutical company “Theraplix (via business at Chimie et Atomistique)” [50]. Credit for ethionamide going to Theraplix is reinforced by information found in the book Drug Discovery: A History: “The Theraplix company in Paris subsequently introduce ethionamide, but it is now rarely used” [51]. Theraplix was taken over by Rhône Poulenc in 1956, and this is now part of Sanofi [52]. |
|  | Gatifloxacin | Gfx | Kyorin Pharmaceutical Co., Ltd. | Patent Number 5880283 [53]. |
|  | Isoniazid | H | Hoffman LaRoche | The earliest patent for Isoniazid (formerly isonicotinylhydrazine) is by Hoffman La Roche in 1952 [54]. Hoffman La Roche’s holding company is Roche Holding, AG. |
|  | Kanamycin | Km | Bristol Myers Squibb | Patent Number EP 0525588 [55]. |
|  | Levofloxacin | Lfx | Daiichi Sankyo | Levofloxacin was developed by Daiichi and approved by the FDA in 1996 [56]. |
|  | Moxifloxacin | Mfx | Bayer | Patent Number 5607942 [57]. |
|  | Ofloxacin | Ofx | Daiichi Sankyo | Ofloxacin was first patented by Daiichi Pharmaceutical Co., Ltd. in 1980 [58]. Daiichi has since merged with Sankyo Co., Ltd. to form Daiichi Sankyo Co., Ltd. |
|  | PAS | PAS | Pfizer | Jorgen Lehmann developed PAS (4-aminosalicylic acid) while working with Ferrosan [59]. Ferrosan received the first patent for PAS in 1948 [60]. Ferrosan is now part of Pfizer [61]. |
|  | Pyrazinamide | Z | Pfizer | The earliest patent for pyrazinamide was granted in 1954 (filed in 1952) by the American Cyanamid Company, which was merged with American Home Products in 1994 [62]. This subsequently changed its name to the Wyeth Corporation, and then merged with Pfizer in 2009. |
|  | Rifampicin | R | Sanofi | The earliest patent for Rifampicin was filed in 1965 by Gruppo Lepetit [63], a subsidiary of Sanofi [64]. |
|  | Streptomycin | S | Merck | Reference: Rutgers developed with Merck funding and they got a license for marketing the drug Patent Number 2449866 [65, 66]. |
| Malaria | Artemether-Lumefrantine | AL | Novartis | Novartis first patented AL [67]. It sells the drug under the trade name Coartem [68]. |
|  | Artesunate + Amodiaquine | AS + AQ | Sanofi | Robert Sauerwein credits Sanofi for ASAQ in multiple articles [69]. Richerd Haynes also credits Sanofi for ASAQ [70]. DNDi also credits Sanofi for ASAQ [71]. The first patent for ASAQ seems to be one filed in 1988 by Hoechst. The patent concerns “combinations of the antimalarials artemisinin, dihydroartemisinin, arteether, artemether, artesunate or other artemisinin derivatives with one or more of the antimalarials chloroquine, 10-O-methylfloxacrine, quinine, mefloquine, amodiaquine, pyrimethamine, sulfadoxine and primaquine. Synergistic effects are achieved on treatment of mammals, including humans, with subcurative doses of the individual substances.” Hoechst is now part of Sanofi [72]. |
|  | Artesunate + Mefloquine | AS + MQ | Public Sector – Military | According to Doctors Without Borders, “ASMQ was developed in the public sector, will not be patented and therefore can be available as a low cost generic immediately” [73]. |
|  | Artesunate + Sulfadoxine-Pyrimethamine | AS + SP | Sanofi | The first patent for AS+SP seems to be one filed in 1988 by Hoechst AG. The patent concerns “combinations of the antimalarials artemisinin, dihydroartemisinin, arteether, artemether, *artesunate* or other artemisinin derivatives with one or more of the antimalarials chloroquine, 10-O-methylfloxacrine, quinine, mefloquine, amodiaquine, pyrimethamine, sulfadoxine and primaquine. Synergistic effects are achieved on treatment of mammals, including humans, with subcurative doses of the individual substances” [73]. Hoechst is now part of Sanofi [73]. We originally credited Advacare for claiming credit |
|  | Dihydroarteminisin-Piperaquine | DHA-PPQ | Chongqing Tonghe Pharmaceutical Co. Ltd | A patent for Dihydroartemisinin-Piperaquine for use in treatment of Malaria was first applied for by Chongqing Tonghe Pharmaceutical Co., Ltd in 2000 (US Patent issued 2010) [74]. |

References

1. Udwadia ZT. MDR, XDR, TDR tuberculosis: ominous progression. Thorax. 2012 Apr;67(4): 286-8.

2. Institute for Health Metrics and Evaluation. Botswana global burden of disease study 2010 (GBD 2010). Seattle: Global Health Data Exchange; 2010. Available: <http://ghdx.healthmetricsandevaluation.org/country_profiles>. Accessed 5 Oct 2013.

3. World Health Organization. Tuberculosis (TB), Data for global tuberculosis control 2011. Country data, case notifications. Geneva: World Health Organization; 2011. Available: <http://who.int/tb/country/data/download/en/index.html>. Accessed 20 Aug 2012.

4. World Health Organization. Global tuberculosis report 2013. Geneva: World Health Organization; 2013.

5. World Health Organization. Multidrug-resistant tuberculosis (MDR-TB) 2013 update. Geneva: World Health Organization; 2013. Available: http://www.who.int/tb/challenges/mdr/MDR_TB_FactSheet.pdf. Accessed 1 Apr 2014.

6. World Health Organization. 2007-2008 XDR & MDR tuberculosis global response plan. Geneva: World Health Organization; 2008. Available: <http://www.who.int/tb/xdr/xdr_mdr_factsheet_2007_en.pdf>. Accessed 1 Jan 2013.

7. World Health Organization. Implementing the WHO stop TB strategy: a handbook for national tuberculosis control programmes. Geneva: World Health Organization; 2008.

8. World Health Organization. TB/HIV facts 2011. Geneva: World Health Organization; 2011. Available: <http://www.who.int/tb/challenges/hiv/factsheet_hivtb_2011.pdf>. Accessed 20 Aug 2012.

9. World Health Organization. Management of MDR-TB: a field guide. Geneva: World Health Organization; 2009. Available: http://apps.who.int/iris/bitstream/10665/44163/1/9789241547765_eng.pdf. Accessed 18 Sep 2015.

10. Shah SN, Wright A, Bai GH, Barrera L, Boulahbal F, Martín-Casabona N, et al. Worldwide emergence of extensively drug-resistant tuberculosis. Emerg Infect Dis. 2007 Mar;13(3): 380-87. Available: <http://www.ncbi.nlm.nih.gov/pmc/articles/PMC2725916/>. Accessed 24 Dec 2012.

11. Mphahlele M, Syre H, Valvatne H, Stavrum R, Mannsåker T, Muthivhi T, et al. Pyrazinamide resistance among South African multidrug-resistant mycobacterium tuberculosis isolates. J Clin Microbiol. 2008 Oct;46(10): 3459-3464. Available: <http://jcm.asm.org/content/46/10/3459.full>. Accessed 4 Jan 2013.

12. Mitnick CD, Shin SS, Seung KJ, Rich ML, Atwood SS, Furin JJ, et al. Comprehensive treatment of extensively drug-resistant tuberculosis. New Eng J Med. 2008 Aug 7;359(6): 563-74. Available: http://www.ncbi.nlm.nih.gov/pmc/articles/PMC2673722/. Accessed 18 Sep 2015.

13. World Health Organization. Antiretroviral medicines in low- and middle-income countries: forecasts of global and regional demand for 2012-2015. Geneva: World Health Organization; 2013. Available: <http://www.who.int/hiv/pub/amds/2013forecast_report/en/>. Accessed 10 Jan 2013.

14. World Health Organization. Estimated antiretroviral therapy coverage among children. Geneva: World Health Organization; 2010. Available: <http://apps.who.int/gho/indicatorregistry/App_Main/view_indicator.aspx?iid=2966>. Accessed 5 Oct 2013.

15. Institute for Health Metrics and Evaluation. Global burden of disease study 2010 results 1990-2010. Results by cause, summing Ages 0-14 and 15+, DALYs 2010. Seattle: Institute for Health Metrics and Evaluation; 2013. Available: <http://ghdx.healthmetricsandevaluation.org/country_profiles>. Accessed 5 Oct 2013.

16. World Health Organization. Data on the HIV/AIDS response: antiretroviral therapy coverage. Annex 4. Geneva: World Health Organization; 2010. Available: <http://www.who.int/hiv/data/en/>. Accessed 5 Oct 2013.

17. Joint United Nations Programme on HIV/AIDS. UNAIDS data tables 2011. Geneva: World Health Organization; 2011. Available: <http://www.unaids.org/en/media/unaids/contentassets/documents/unaidspublication/2011/JC2225_UNAIDS_datatables_en.pdf>. Accessed 1 Jan 2013.

18. World Health Organization. Global HIV/AIDS response. Geneva: World Health Organization; 2011. Available: <http://whqlibdoc.who.int/publications/2011/9789241502986_eng.pdf>. Accessed 1 Jan 2013.

19. World Health Organization. Antiretroviral medicines in low- and middle-income countries: usage in 2010 with global and regional demand forecast for 2011 – 2012.  Geneva: World Health Organization; 2010. p. 4.

20. Hughes W. Safety and single-dose pharmacokinetics of abacavir (1592U89) in human immunodeficiency virus type 1-infected children. Antimicrob Agents Chemother. 1999 Mar;43(3): 609-15. Available: http://www.ncbi.nlm.nih.gov/pmc/articles/PMC89168/. Accessed 19 Oct 2015.

21. Barry WD, St-Clair MH. Synergistic combinations of zidovudine, 1592u89 and 3tc. United States patent US 6417191. 2002 Jul 9. Available: <http://www.google.com/patents/US6417191>. Accessed 15 Feb 2013.

22. The Chicago Tribune. FTC approves Glaxo-Wellcome merger. Chicago Tribune. 16 Mar 1995. Available: <http://articles.chicagotribune.com/1995-03-16/news/9503170329_1_glaxo-wellcome-glaxo-plc-wellcome-plc>. Accessed 15 Feb 2013.

23. British Broadcast Company. Profile: Glaxo Wellcome. BBC News. 17 Jan 2000. Available: <http://news.bbc.co.uk/2/hi/business/606752.stm>. Accessed 15 Feb 2013.

24. What We Do. 2015 [cited 15 Feb 2013]. In: ViiV Healthcare Company Website. Viiv Healthcare. Available: https://www.viivhealthcare.com/what-we-do.aspx. [Accessed](http://www.viivhealthcare.com/about-us/heritage.aspx?sc_lang=en) 25 Oct 2015.

25. Reuters T. Patent landscape report on Atazanavir. Geneva: World Intellectual Property Organization; 2011. p. 6. Available: h[ttp://www.wipo.int/export/sites/www/freepublications/en/patents/946/wipo_pub_946_2.pdf](http://www.wipo.int/export/sites/www/freepublications/en/patents/946/wipo_pub_946_2.pdf). Accessed 12 Jan 2013.

26. Landon IP. Patent landscape report on ritonavir. Geneva: World Intellectual Property Organization; 2011. Available: <http://www.wipo.int/patentscope/en/programs/patent_landscapes/reports/ritonavir.html>. Accessed 12 Jan 2013.

27. Didanosine-Videx. [cited 8 Mar 2013]. In: The Medical Dictionary [internet]. Available: <http://the-medical-dictionary.com/didanosine.htm>. Accessed 19 Oct 2015.

28. Young SD, Britcher SF, Payne LS, Tran LO, Lumma WC. Benzoxazinones as inhibitors of HIV reverse transcriptase. Unites States patent US 5519021. 21 May 1996. Available: http://www.google.com/patents/US5519021. Accessed 8 Mar 2013.

29. Liotta DC, Schinazi RF, Choi WB. Method for the synthesis, compositions, and case of 2’-Deoxy-5-Fluoro-3’-Thiacytidine and related compounds. United States patent US 5814639. 29 Sep 1998. Available: <http://www.google.com/patents/US5814639>. Accessed 8 Mar 2013.

30. Gilead Sciences, Emory University, Royalty Pharma. Gilead sciences and royalty pharma announce $525 million agreement with Emory University to purchase royalty interest for emtricitabine. Atlanta: Emory University; 2005. Available: <http://www.emory.edu/news/Releases/emtri/>. Accessed 8 Mar 2013.

31. Belleau B, Nguyen-Ba N. 2-substituted-5-substituted-1,3-oxathiolanes with antiviral properties. United States patent US 5047407. 10 Sep 1991. Available: http://www.google.com/patents/US5047407. [Accessed 7 Mar 2013.](http://www.google.com/patents/US5047407)

# 32. CBS News. BioChem pharma agrees to $13-billion merger. CBC News. 11 Dec 2000. Available: <http://www.cbc.ca/news/business/story/2000/12/11/biochem001211.html>. Accessed 7 Mar 2013.

33. Kempf DJ, Norbeck DW, Sham HL, Zhao C. Retroviral protease inhibiting compounds. United States patent US5541206. 130 Jul 1996. Available: http://www.google.com/patents/US5541206. Accessed 2 Mar 2013.

34. Dressman BA, Fritz JE, Hammond M, Hornback WJ, Kaldor SW, Kalish VJ, et al. HIV protease inhibitors. United States patent US 5484926. 16 Jan 1996. Available: http://www.google.com/patents/US5484926. Accessed 4 Mar 2013.

35. History. [cited 4 Mar 2013]. In: Agouron Institute Website [internet]. Agouron Institute. Available: http://agi.org/about/history/. Accessed 19 Oct 2015.

36. Christmann A, Heinrich S. Process for the preparation of nevirapine. European patent EP 0667348. 10 May 2000. Available: <http://www.google.com/patents/EP0667348B1> Accessed 5 Mar 2013.

37. Skonezny PM, Eisenreich E, Stark DR, Boyhan BT, Baker S R. Process for large-scale preparation of 2',3'-didehydro-2',3'-dideoxynucleosides. United States patent US 5539099. 23 Jul 1996. Available: http://www.google.com/patents/US5539099. Accessed 8 Mar 2013.

38. Bischofberger NW. PMPA preparation. United States patent US 5733788. 31 Mar 1998. Available: <http://www.google.com/patents/US5733788>. Accessed 28 Jan 2013.

39. Roberts T G, Evans P. Crystalline oxathiolane derivatives. United States patent US 5905082. 18 May 1999. Available: <http://www.google.com/patents/US5905082>. Accessed 7 Mar 2013.

40. Naito T, Okumura J, Hoshi H. Novel penicillins. Lines 53-56: antibiotics, bactericides, pseudomonas. United States patent US 4206116. 3 Jun 1980. Available: <http://www.google.com/patents/US4206116>. Accessed 8 Mar 2013.

41. Kawaguchi H, Naito T, Nakagawa S. Antibiotic derivatives of kanamycin. United States patent US 3781268. Available: <http://www.google.com/patents/US3781268>. Accessed 8 Mar 2013.

42. Kraus C, Barry C, Doan B. Aerosolized capreomycin for inhibition of pulmonary tuberculosis. United States patent US 20070128124. 7 Jun 2007. Available: <http://www.google.com/patents/US20070128124>. Accessed 8 Mar 2013.

43. Shull GM, Routien JB, Finlay AC. Cycloserene and production thereof. United States patent US 2773878. 5 Mar 1952. Available: <http://www.google.com/patents/US2773878>. Accessed 8 Mar 2013.

44. Singh B. Synthesis of ethambutol. United States patent US 3944618. 16 Mar 1976. Available: http://www.google.com/patents/US3944618. Accessed 5 Feb 2013.

45. Federal Trade Commission. FTC settles charges with American Cyanamid; agency alleged company had fixed prices and restricted competition. Federal Trade Commission Press Releases. 30 Jan 1997. Available: <http://www.ftc.gov/news-events/press-releases/1997/01/ftc-settles-charges-american-cyanamid-agency-alleged-company-had>. Accessed 4 Feb 2013.

46. Peterson M. American Home is changing name to Wyeth. The New York Times. 11 Mar 2002. Available: http://www.nytimes.com/2002/03/11/business/american-home-is-changing-name-to-wyeth.html. Accessed 4 Feb 2013**.**

47. About Pfizer. 2002-2015 [cited 4 Feb 2013]. In: Pfizer Company Website [internet]. Pfizer. Available: http://www.pfizer.com/about. Accessed 19 Oct 2015.

48. Liberman D. Certain 2-substituted isonicotinic thioamides. United States patent US 2901488. 25 Aug 1959. Available: <http://www.google.com/patents/US2901488>. Accessed 15 Feb 2013.

49. Redel J. New derivatives of trioxo-2,4,6-piperidine and the process of preparing same. United States patent US 3048590. 7 Aug 1962. Available: http://www.google.com/patents/US3048590. Accessed 15 Mar 2013.

50. Académie française. François Albert Buisson. Paris: Academie Francaise Institute. Available: <http://translate.google.com/translate?hl=en&sl=fr&u=http://www.academie-francaise.fr/les-immortels/francois-albert-buisson&prev=/search%3Fq%3DFran%25C3%25A7ois%2BAlbert-Buisson%2Btheraplix%26hl%3Den%26safe%3Doff%26biw%3D1190%26bih%3D547&sa=X&ei=lnxDUamuBo_G4AOZ-oDgCg&ved=0CFAQ7gEwBA>. Accessed 15 Mar 2013.

51. Sneader W. Drug discovery: a history. West Sussex: John Wiley & Sons, Ltd; 2005. p. 396. Available: http://books.google.com/books?id=jglFsz5EJR8C&pg=PA396&lpg=PA396&dq=Theraplix+ethionamide&source=bl&ots=DE1f4A8V3c&sig=cnSsHTjdDZHPBLpEoYnslJH7F2g&hl=en&sa=X&ei=13lDUbObApK04AONloGIAg&ved=0CGAQ6AEwCQ#v=onepage&q=Theraplix%20ethionamide&f=false. Accessed 15 Mar 2013.

52. Sanofi. Rhône Poulenc. 2013 [cited 15 Mar 2013]. In: Sanofi Company Website [internet]. Sanofi. Available: <http://en.sanofi.com/history/ajax/en_rhone_poulenc.html>. Accessed 19 Oct 2015.

53. Matsumoto T, Hara M, Miyashita K, Kato Y. 8-Alkoxyquinolonecarboxylic acid hydrate with excellent stability and process for producing the same. United States patent US 5880283. 9 Mar 1999. Available: <http://www.google.com/patents/US5880283>. Accessed 8 Mar 2013.

54. Fox HH. Compositions for combating tuberculosis. United States patent US 2596069. 6 May 1952. Reissued 15 Feb 1955. Available: [http://www.google.com/patents/US2596069](http://www.google.com/patents/US2596069%20). Accessed 21 Feb 2013.

55. Furumai T, Hatori M, Kakushima M, Ikeda C, Saitoh K, Kobaru S. Production of pradimicin antibiotics by actinomadura strain. European patent EP 0525588. 3 Feb 1993. <http://www.google.com/patents/EP0525588A2>. Accessed 8 Mar 2013.

56. Puig TS, Bessa B J. Process for the preparation of an antibacterial quinolone compound. European Patent EP 1939206 A1. 22 Dec 2006. Available: <http://www.google.com/patents/EP1939206A1?cl=en>. Accessed 28 Feb 2013.

57. Peterson U, Schenke T, Krebs A, et al. 7-(1-pyrrolidinyl)-3-quinolone- and - naphthyridone-carboxylic acid derivatives as antibacterial agents and feed additives. United States patent US 5607942. 4 Mar 1997. Available: <http://www.google.com/patents/US5607942>. Accessed 3 Mar 2013.

58. Janssen-Ortho Inc. v. Novopharm Limited. 2006 FC 1234. Paragraph 24. Database: Federal Court Decisions [internet]. Accessed: http://decisions.fct-cf.gc.ca/en/2006/2006fc1234/2006fc1234.html. Accessed 4 Feb 2013.

59. Ryan F. Tuberculosis: the greatest story never told. Sheffield: Swift Publishers Ltd.; 1992.

60. Karl-Gustaf R. Process for producing a 4-amino salicylic acid. United States patent US 2445242. 13 Jul 1948. Available: <https://www.google.com/patents/US2445242>. Accessed 8 Jan 2014.

61. About Us. 2015 [cited 8 Jan 2014]. In: Ferrosan Medical Devices Website [internet]. Soeborg: Ferrosan Medical Devices A/S. Available: http://www.ferrosanmedicaldevices.com/Ferrosan2012/About-Us.aspx#.ViFL-Gve88k. Accessed 19 Oct 2015.

62. James HW, Kushner S. Tubekculostatic Agent. United States patent US 2677641. 4 May 1954. Available: <http://www.google.com/patents/US2677641>. [Accessed 1 Nov 2012.](http://www.google.com/patents/US2677641)

63. Nicola M, Piero S. Derivatives of rifamycin. United States patent US 3342810. 19 Sep 1967. Available: <http://www.google.com/patents/US3342810>. Accessed 5 Jan 2013.

64. Our Company. 3 Jul 2015 [cited 5 Jan 2013]. In: Sanofi Company Website [internet]. Sanofi. Available: [http://en.sanofi.com/our_company/worldwide/italy.aspx](http://www.google.com/url?q=http%3A%2F%2Fen.sanofi.com%2Four_company%2Fworldwide%2Fitaly.aspx&sa=D&sntz=1&usg=AFQjCNHKtqbbzDjOW0bR6GYcEXRLRSDMnA). Accessed 19 Oct 2015.

65. Waksman SA, Albert S. Streptomycin and process of reparation. United States patent US 2449866. 21 Sep 1948. Available: <http://www.google.com/patents/US2449866>. Accessed 6 Mar 2013.

66. Kingston W. Streptomycin, Schatz v. Waksman, and the balance of credit for discovery. J Hist Med Allied Sci. 2004 Jul;59(3): 441-62. p. 450. Available: <http://www.ncbi.nlm.nih.gov/pubmed/15270337>. Accessed 18 Feb 2015.

67. Allmendinger T, Wernsdorfer WH. Benflumetol derivatives, intermediates thereof and their use against parasitical protozoa and trematodes. European patent EP 1089961. 11 Apr 2001. Available: <https://www.google.com/patents/EP1089961A1>. Accessed 8 Mar 2014.

68. The Novartis malaria initiative: lessons from a dramatic decade. 17 Nov 2011 [cited 13 Mar 2014]. In: Novartis Newsroom [internet]. Novartis AG. Available: http://www.malaria.novartis.com/newsroom/featured-news/2011-04-malaria-initiative-1.shtml. Accessed 19 Oct 2015.

69. Bousema JT, Schneider P, Gouagna LC, Drakeley CJ, Tostmann A, Houben R, et al. Moderate effect of artemisinin-based combination therapy on transmission of plasmodium falciparum. Arlington: Infectious Diseases Society of America; 2006. p. 1152. Available: <http://jid.oxfordjournals.org/content/193/8/1151.full.pdf>. Accessed 23 Nov 2012.

70. Haynes RK. Artemisinins: remarkable antimalarial drugs, usages, and problems, and a new derivative-artemisone. International Centre for Science and High Technology, AREA Science Park: United Nations Industrial Development Organization Seminar on Advanced Design and Development of Potential Drugs Against Malaria; 2009 Mar 12; Trieste, Italy.

71. Articles. Database: Drugs for Neglected Diseases initiative [internet]. Available: <http://dndi.org/component/content/article/347-about-dndi/key-accomplishments/1212-malaria.html?highlight=YToyOntpOjA7czo0OiJhc2FxIjtpOjE7czo2OiJzYW5vZmkiO30>. Accessed 22 Nov 2012.

72. Chatterjee DK, Venugopalan B, Lal B, De SNJ, Rupp RH. Drug mixture for prophylaxis and therapy of malaria. European patent EP0290959. 17 Nov 1988. Available: <http://www.google.com/patents/EP0290959A2?cl=en&dq=artesunate+amodiaquine&hl=en&sa=X&ei=DhfCUIqFLMeC0QGYxoCoBw&ved=0CDcQ6AEwAA>. Accessed 16 Nov 2012.

73. Doctors Without Borders/Médecins Sans Frontières. MSF welcomes new fixed-dose combination against malaria. 18 Apr 2008 [cited 3 Dec 2012]. In: Doctors Without Borders/Médecins Sans Frontières Press Releases [internet]. Available: http://www.doctorswithoutborders.org/news-stories/press-release/msf-welcomes-new-fixed-dose-combination-against-malaria. Accessed 19 Oct 2015.

74. Li G, Song J. Composition containing artemisinin for treatment of malaria. US Patent Number 7851512. 14 Dec 2010. Available: <http://www.google.com/patents/US7851512>. [Accessed 1 Jan 2013.](http://www.google.com/patents/US7851512)
